# Small Area Variation of Adherence to Clinical Recommendations: An Example from Switzerland

**DOI:** 10.1177/23333928221097741

**Published:** 2022-05-11

**Authors:** Oliver Gruebner, Wenjia Wei, Agne Ulyte, Viktor von Wyl, Holger Dressel, Beat Brüngger, Caroline Bähler, Eva Blozik, Matthias Schwenkglenks

**Affiliations:** 1 Epidemiology, Biostatistics, and Prevention Institute, 27217 University of Zurich, Zurich, Switzerland; 2 Department of Geography, 27217 University of Zurich, Zurich, Switzerland; 3 Department of Health Sciences, 60558 Helsana Insurance Group, Zurich, Switzerland; 4 Institute of Primary Care, 27217 University of Zurich, Zurich, Switzerland

**Keywords:** access to health care, health care utilization, clinical guidelines, unwarranted variation, spatial analysis

## Abstract

**Background:**

Unwarranted variation in healthcare utilization can only partly be explained by variation in the health care needs of the population, yet it is frequently found globally. This is the first cross-sectional study that systematically assessed geographic variation in the adherence to clinical recommendations in Switzerland. Specifically, we explored 1) the geographic variation of adherence to clinical recommendations across 24 health services at the sub-cantonal level, 2) assessed and mapped statistically significant spatial clusters, and 3) explored possible influencing factors for the observed geographic variation.

**Methods:**

Exploratory spatial analysis using the Moran’s I statistic on multivariable multilevel model residuals to systematically identify small area variation of adherence to clinical recommendations across 24 health services.

**Results:**

Although there was no overall spatial pattern in adherence to clinical recommendations across all health care services, we identified health services that exhibited statistically significant spatial dependence in adherence. For these, we provided evidence about the locations of local clusters.

**Interpretation:**

We identified regions in Switzerland in which specific recommended or discouraged health care services are utilized less or more than elsewhere. Future studies are needed to investigate the place-based social determinants of health responsible for the sub-cantonal variation in adherence to clinical recommendations in Switzerland and elsewhere over time.

## Strength and Limitations

- Only few studies assessed systematic small area geographic variation of effective (clinically recommended) and inappropriate (clinically discouraged) care. This is the first study that systematically assessed geographic variation of effective and inappropriate care in Switzerland across 24 health services.
- Information on the small area spatial patterning of effective and inappropriate care helps improve locally targeted health care policy, health care utilization incentives, or campaigns directed toward hospitals, physicians, or patients. Yet, national or state-level (in Switzerland: cantonal-level) statistics are utilized for monitoring healthcare system performance. This paper highlights that spatially fine-grained healthcare system performance monitoring is necessary to further improve health outcomes rather than exclusively evaluating national or state-level statistics, which is still common practice in Switzerland and comparable settings worldwide.
- Although our data is from one of the largest available datasets in Switzerland, future studies should aim at combining data from various insurance companies to make the dataset as representative as possible at the MS level of analysis.
- Furthermore, Swiss health insurance claims data do not contain diagnosis information, patient and provider preferences, or provider characteristics. Therefore, future studies should triangulate health insurance data also with other available data while acknowledging legal and ethical concerns to investigate the geographic variation of adherence across time.

## Introduction

Large variation in the utilization of healthcare services is regularly found across hospitals, healthcare providers, and geographic regions.^1–3^ However, the ubiquity and persistence of such variation can only partly be explained by variation in the health care needs of the population. Inappropriate and hence unwarranted variation may be due to a multitude of factors, including healthcare system-related factors such as the availability and accessibility of physicians and healthcare facilities and also patient socio-demographics and clinical characteristics.^2–5^ Another important factor is variation in the implementation of clinical recommendations, which cannot be fully evaluated with national or state-level (in Switzerland: cantonal-level) statistics.^1^ Although such analyses are crucial for monitoring overall healthcare system performance, they do not consider the local response to clinical recommendations for or against specific health care services. Focusing on smaller geographic units offers opportunities to explore whether health policy measures or clinical recommendations are uniformly or diversely implemented within the higher-level entities (eg, states or cantons) that are responsible for formulating health policies.

In Switzerland, health care planning and delivery is, in principle, organized at the level of cantons.^5^ However, the mountainous topography of Switzerland may create heterogeneity of health policy implementation or access to specialist care even within larger cantons, for example in more remote areas (eg, canton of Valais), or regions speaking a different language than the main cantonal or regional centre (eg, in bilingual cantons such as Fribourg or Bern). On the other hand, there may also be spill-over effects, for example, by larger, tertiary hospitals caring for, and influencing treatment practices of, larger, cross-cantonal regions.

Only a few studies have assessed sub-cantonal geographic variation in Switzerland in under supply of effective health care which has been clinically recommended, and over supply of inappropriate care which has been clinically discouraged. As reported in Wei *et al*,^6^ our group found that health care utilization rates varied considerably across 24 Swiss healthcare services and 106 sub-cantonal (mobilité spatiale - MS) regions. Our study^6^ found that having a higher deductible level was consistently associated with lower utilization of most health care services, whether recommended or discouraged, and that managed care tended to be associated with better guideline adherence. However, this study did not investigate systematic geographic patterns of health care utilization of effective and inappropriate care. Hence, we do not know the extent to which neighbouring sub-cantonal regions in Switzerland share similar adherence patterns as indicated by local spatial dependence or spatial interaction processes. In addition, the sub-cantonal regions in Switzerland that exhibit relatively low or high levels of adherence to clinical recommendations, with respect to the national average, have not been identified. Information on the spatial patterning of adherence can help direct improvement and indicate guidance needed, such as locally targeted health care policy, health care utilization incentives, or campaigns directed toward hospitals, physicians, or patients.

In this study, we investigate whether specific regions consistently exhibit higher or lower adherence to clinical recommendations, thus indicating a possible need for more targeted health policy measures. Our specific aims were to 1) explore the geographic variation of adherence to clinical recommendations across 24 health services in Switzerland, to 2) assess and map statistically significant spatial clusters of relatively good (hotspots) or relatively poor adherence to clinical recommendations (cold spots), and to 3) qualitatively explore possible influencing factors for the observed sub-cantonal geographic variation. To this end, we constructed a framework to describe the observed variation and to generate further hypotheses about specific drivers of clinical recommendation variation.

## Methodology

### Selection of Clinical Recommendations

We focused mostly on primary healthcare services for major non-communicable diseases, based on a systematic approach to selection described earlier.^6,7^ Briefly, we considered recommendation statements from clinical practice guidelines of relevant international and Swiss medical societies. We only considered recommendation statements that were relevant to Switzerland and the adherence to which could be assessed from Swiss health insurance claims data. Table 1 details the selected 24 health care services and the respective clinical recommendations.

**Table 1.** Groups of Health Care Services and Descriptions.

Group	Health Care Service	Approximated recommendation applied in the study	Study population	Recommendation	Recommended
**Screening**	Colon cancer screening	Colonoscopy/ year	Anyone 50–69 years old	Colonoscopy should be done every 10 years for people 50–69 years old.	YES
	Breast cancer screening	Mammography/ year	50–74 years old women	Mammography should be done every 2 years for 50–74 years old women.	YES
	Prostate cancer screening	PSA testing/ year	50–70 years old men	Routine prostate cancer screening with PSA testing is not recommended.	NO
	Osteoporosis screening	DXA/ year	People over 60 and with risk factors of spontaneous fractures	DXA densitometry is recommended for patients with spontaneous fractures or increased risk of them.	YES
**Diagnosis**	DM: HbA1c test	HbA1c test twice/ year	Adult drug-treated diabetes patients	HbA1c test should be done for diabetes patients at least twice a year.	YES
	DM: kidney exam	Albuminuria and serum creatinine tests/ year	Adult drug-treated diabetes patients	Albuminuria and serum creatinine tests should be done for diabetes patients at least once a year.	YES
	DM: LDL test	LDL test/ year	Adult drug-treated diabetes patients under 75 years old	LDL test should be done for diabetes patients at least once a year.	YES
	DM: eye examination	Ophthalmologist visit/ year	Adult drug-treated diabetes patients	Eye exam should be performed for diabetes patients at least once a year.	YES
	TSH screening	TSH test without T3 and T4 tests on the same day	Adults without thyroid disease and receiving TSH test	TSH should be measured as an initial screening test for hypo/hyperthyroidism, while T3 and T4 test should follow if TSH is abnormal.	YES
	POCR	Outpatient POCR up to 2 months before surgery	Adult patients with inpatient surgical procedures	Routine chest radiography is not recommended before surgery.	NO
**Primary prevention**	Influenza vaccination	Influenza outpatient vaccination/ year	People over 65 years old or with a specified chronic condition	People over 65 years old and patients with chronic conditions, specified by Federal Office of Public Health, should be vaccinated against influenza every year.	YES
**Secondary prevention**	AMI: aspirin	Aspirin prescription within 2 weeks after AMI	Adult patients with AMI	All AMI patients should take aspirin long-term.	YES
	AMI: statin	High-dose statin prescription within 2 weeks after AMI	Adult patients with AMI	All AMI patients should get statins long-term.	YES
	AMI: beta-blocker	Beta-blocker prescription within 2 weeks after AMI	Adult patients with AMI	All AMI patients with heart failure or impaired function should get beta-blockers long-term.	YES
	AMI: ACE/ARB	ACE or ARB antihypertensive medication prescription within 2 weeks after AMI	Adult patients with AMI	All AMI patients with heart failure or impaired function should get ACE or ARB antihypertensive medication long-term.	YES
	AMI: P2Y12 inhibitors	P2Y12-inhibiting antiplatelet drug prescription within 2 weeks after AMI	Adult patients with AMI	All AMI patients should get P2Y antiplatelet drugs for at least 1–12 months according to the bleeding risk profile and AMI treatment.	YES
	PPI with NSAID	PPI prescription within 1 month or up to 3 months before initial long-term NSAID prescription	Adult patients with a cumulative NSAID prescription of >8 weeks at maximal dose	Patients taking long-term NSAID and with risk factors for gastric ulcer should also take PPI.	YES
	PAD: statin	Prescription of statins within 3 months after PAD identification	Adult patients undergoing diagnostic or treatment procedures for PAD	Statins are recommended for all patients with PAD.	YES
	Afib: anticoagulation	Oral anticoagulation prescription within 2 weeks after Afib identification	Adult patients with atrial fibrillation diagnosis and additional risk factors	All patients with Afib should be prescribed oral anticoagulation for embolic events prevention according to the CHA_2_DS_2_-VASc score.	YES
	GCC with new DMARD	GCC prescription within 1 month or up to 3 months before DMARD prescription	Adult patients with a new DMARD prescription by a rheumatologist	Short-term glucocorticoids should be taken with newly prescribed DMARD.	YES
**Treatment**	Benzodiazepines	Cumulative prescription of BZD for >8 weeks/ year	Anyone over 65 years old	Long-term use of BZD and other hypnotics is discouraged for old patients.	NO
	Proton pump inhibitors	Cumulative prescription of PPI or H2 for >8 weeks/ year	Adults receiving PPI or H2 drugs	PPI should not be used at maximal dose for prolonged periods of time.	NO
	Outpatient procedures	Specified surgical procedures done in the outpatient setting	Adult patients with specified surgical procedures (either as in- or outpatient)	If none of the special conditions apply, certain surgical procedures should be done in the outpatient setting.	YES
	Caesarean section	Caesarean section (C-section)	Women giving birth without absolute indications for C-section	C-section should not be performed unless absolute or relative indications are present.	NO

ACE/ARB – angiotensin-converting enzyme inhibitors or angiotensin II receptor blockers, Afib – atrial fibrillation, AMI – acute myocardial infarction, BZD – benzodiazepines, DM – diabetes mellitus, DMARD – disease-modifying antirheumatic drug, DXA – Dual-energy x-ray absorptiometry, GCC – glucocorticosteroid drugs, HbA1c – Glycated hemoglobin, H2 – histamine H_2_ receptor antagonists, LDL – low density lipoprotein, NSAID – nonsteroidal anti- inflammatory drugs, PAD – peripheral artery disease, POCR – preoperative chest radiography, PPI – proton pump inhibitors, PSA – Prostate-specific antigen, TSH – thyroid-stimulating hormone, T3 – triiodothyronine, T4 - thyroxine. See Ulyte *et al* ^14^ for approximation of clinical recommendation in eg, screening participation studies.

### Study Design and Geographic Level of Analysis

We used an ecological and cross-sectional study design taking the spatial mobility regions (mobilité spatiale - MS) as the geographic units of analysis (N = 106).^8^ MS regions are defined by the Swiss Federal Statistical Office and used as intermediate-size units of analysis for scientific and regional policy purposes.

### Patient and Public Involvement

No patient involved.

### Dataset and Outcome Variables

Our study is based on claims data that covered mandatory health insurance provided by the Helsana group in Switzerland to around 15% of the Swiss population (ie, 1.2 million enrolees) for the year 2014. The outcome variables used for this study were MS-level (level-2) regression residuals that were found in a previous multilevel study.^6^ Briefly, in this previous study, adherence to the clinical recommendations accompanying the above mentioned 24 health care services were modelled in a set of multivariable two-level logistic regression models (MS region as the second level), adjusted for socio-demographic, clinical, and health insurance-related characteristics, and resident MS region. These residuals from the overall mean indicate the unexplained, small geographic unit-level deviation in adherence to clinical recommendations (based on health care utilization), which we analysed to identify spatial clusters and draw conclusions on the similarity or dissimilarity of the small geographic units (MS regions).

For the current study, we organized the MS-level residuals of the 24 health care services into five conceptual groups: Screening (N = 4), diagnosis (N = 6), primary prevention (N = 1), treatment (N = 4), and secondary prevention (N = 9). Descriptive statistics for all MS-level residuals of the multilevel regression models are shown in Table 2.

**Table 2.** Descriptive Statistics for all Services Used in the Study. Mobilité Spatiale - MS Level (Level-2) Residuals from Multilevel Logistic Regression Models.

Group	Health Care Service	Min.	Pctl. (25)	Median	Pctl. (75)	Max	St. Dev
Screening	Colon cancer screening	−0.305	−0.076	−0.006	0.067	0.265	0.117
	Breast cancer screening	−0.453	−0.101	0.018	0.115	0.431	0.171
	Prostate cancer screening	−0.604	−0.172	−0.009	0.158	0.590	0.215
	Osteoporosis screening	−0.232	−0.057	−0.009	0.071	0.244	0.097
Diagnosis	DM: HbA1c test	−0.428	−0.126	0.011	0.108	0.426	0.172
	DM: kidney exam	−0.422	−0.099	−0.016	0.088	0.501	0.153
	DM: LDL test	−0.403	−0.077	−0.003	0.078	0.420	0.152
	DM: eye examination	−0.741	−0.104	0.0003	0.100	0.624	0.208
	TSH screening	−1.303	−0.197	0.076	0.269	0.891	0.396
	POCR	−0.461	−0.108	−0.010	0.119	0.352	0.167
Primary prevention	Influenza vaccination	−0.536	−0.128	0.010	0.151	0.515	0.179
Secondary prevention	AMI: aspirin	−0.122	−0.017	0.002	0.015	0.111	0.031
	AMI: statin	−0.459	−0.066	0.019	0.073	0.307	0.127
	AMI: beta-blocker	−0.240	−0.045	−0.007	0.031	0.188	0.071
	AMI: ACE/ARB	−0.319	−0.039	0.011	0.048	0.186	0.078
	AMI: P2Y12 inhibitors	−0.146	−0.032	−0.006	0.032	0.139	0.054
	PPI with NSAID	−0.269	−0.055	−0.008	0.073	0.241	0.108
	PAD: statin	−0.244	−0.053	−0.008	0.045	0.291	0.093
	Afib: anticoagulation	−0.484	−0.063	−0.020	0.071	0.280	0.120
	GCC with new DMARD	−0.125	−0.026	0.002	0.021	0.098	0.038
Treatment	Benzodiazepines	−0.345	−0.093	0.005	0.072	0.289	0.130
	Proton pump inhibitors	−0.397	−0.075	0.003	0.078	0.277	0.121
	Outpatient procedures	−0.853	−0.147	0.006	0.149	0.769	0.268
	Caesarean section	−0.272	−0.061	−0.016	0.070	0.407	0.106

### Data Statement

The data used for this study cannot be shared publicly due to patient privacy. The data belong to Helsana (https:// www.helsana.ch/en/helsana-group). Helsana can be contacted to make subsets of the database available for researchers under specific conditions.

### Statistical Analysis

For exploratory spatial data analysis, we used quartile mapping. More specifically, we grouped each of the 24 sets of model residuals into quartiles and determined where values in each of the 24 distributions were found in the upper (>75%) and in the lower quartiles (<25%). We spatially integrated the data with MS regions to map and identify MS regions that were found either in the upper quartile or the lower quartile of the distribution for each set of model residuals. Regions with high positive residuals (upper quartile) are the regions that are relatively better adherent to a clinical recommendation (for a specific healthcare service) after multivariable adjustment. In contrast, high negative residuals (lower quartile) show regions of relatively poor adherence.

In a next step, we assessed the spatial patterning, that is, the degree of spatial clustering of adherence with global spatial autocorrelation analysis using the global Moran’s I statistic (MI). MI values generally range from −1 to 1, with positive values indicating positive spatial autocorrelation (ie, above or below average values of regression residuals clustering across neighbouring MS regions) and negative values indicating negative spatial autocorrelation (ie, dissimilar regression residuals clustering across neighbouring regions). Significant (p-value<0.05) medium (MI> = 0.25) and strong (MI> = 0.5) global spatial autocorrelation statistics were further considered for local spatial cluster analysis.

Local spatial clusters of adherence were analysed using the local Moran’s I statistic. The local Moran’s I statistic is a decomposition of the global Moran’s I and indicates for neighbouring MS regions, where clusters of above average values (high-high pairs of regions), below average values (low-low), or outliers (high-low, low-high) are located. Results of local Moran’s I statistics are presented by mapping statistically significant local clusters exhibiting MS regions with relatively better (high-high) or poor (low-low) adherence to clinical recommendations indicating either hotspots or cold spots, respectively.

Spatial data integration, statistical analyses, and mapping were performed using the R Software and the tmap package in R was used to draw quantile and LISA maps.^9,10^ A p-value of < 0.05 was considered as statistically significant for all analyses.

### Qualitative Framework to Describe Variation Patterns

Given the observation of regions with statistically significant variation (hot spots, cold spots), we considered the following possible causes where there are at least two neighbouring areas with the same type of significant variation (hot spots or cold spots):

1. influence of local health policy and guidelines if they predominantly fall within a political (cantonal) boundary,
2. influence of health care network effects (spatial spill-over effects) if they cross cantonal boundaries and include larger, regionally or nationally important hospitals,
3. topography if they are isolated small area geographic variation in remote areas (although random variation and chance findings cannot be excluded).

Based on this framework, maps and charts were critically reviewed by all authors, and the fit observed patterns with the three scenarios of hypothesized variation was assessed.

## Results

**Figure 1.**
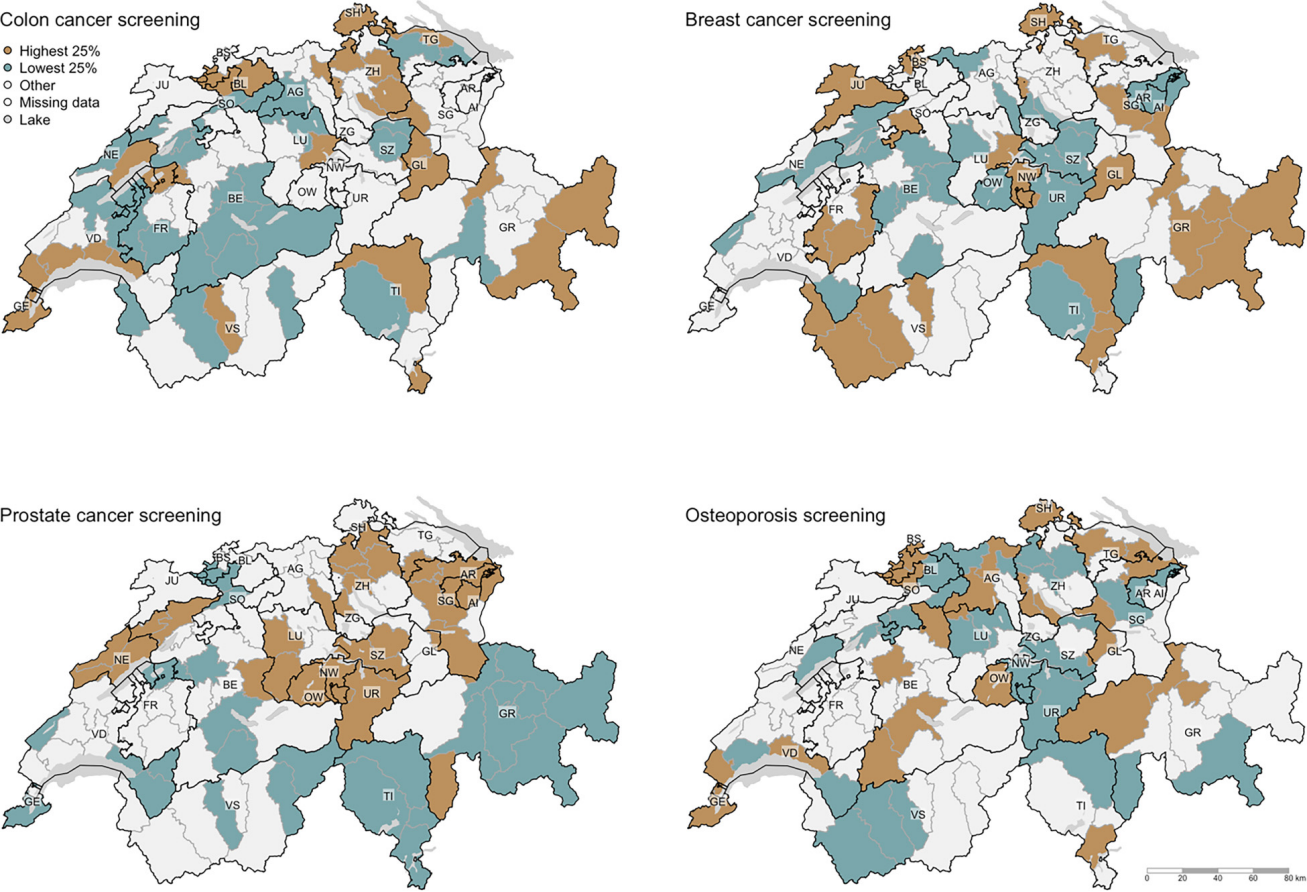
Geographic variation across the studied health care services of screening.

Figure 1 and supplementary Figures S1–4 show quantile map model residuals for adherence to recommendations across the 24 health services in Switzerland considered in this study. The maps visually illustrate that the highest and lowest values (ie, upper or lower quartiles of the residual distribution for a health care service), vary across the services and across regions in Switzerland. For example, Figure 1 (upper left) shows upper quartile residuals for colon cancer screening adherence in MS regions within 16 cantons. In contrast, the lower quartile model residuals were found in MS regions of only 12 cantons. Furthermore, upper quartile model residuals for adherence to breast cancer screening (Figure 1, upper right) were found in MS regions in 14 cantons, while lowest quartile residuals for breast cancer screening adherence were also located in MS regions in 14, but different cantons. The maps for prostate screening (Figure 1, lower left) and osteoporosis screening (Figure 1, lower right) also show geographic variation with different patterns. In general, geographic variation of model residuals of adherence was identified across all 24 health services considered, albeit different MS regions and cantons were affected depending on the health service under consideration (Figure 1, supplementary Figures S1-S4).

In order systematically to quantify the small area geographic variation, we assessed global spatial dependence of adherence across MS regions in Switzerland (Table 3). We found statistically significant, strong spatial clustering only for adherence (ie, non-use) in prostate cancer screening (Moran’ I: .51, p-value: <.001). Statistically significant, medium spatial clustering was found for thyroid stimulating hormone (TSH) testing without T3 and T4 tests on the same day (TSH screening) (.29, <.001), cumulative prescription of benzodiazepines (BZD) for >8 weeks/ year (benzodiazepines) (.27, <.001), glycated hemoglobin (HbA1c) testing twice/ year (DM: HbA1c test) (.25, <.001). Statistically significant small global spatial clustering was found for annual low-density lipoprotein (LDL) testing (DM: LDL test) (.24, <.001) for diabetes patients, for annual albuminuria and serum creatinine testing (DM: kidney exam) (.19,<.01), PPI prescription within one month or up to three months before initial long-term nonsteroidal anti-inflammatory drug (NSAID) prescription (PPI with NSAID) (.19, <.01), high-dose statin prescription within 2 weeks after AMI (AMI: statin) (.16, <.01), specified surgical procedures done in the outpatient rather than inpatient setting (outpatient procedures) (.13, <.05), ophthalmologist visit/ year (DM: eye examination) (.12, <.05), and osteoporosis screening (.10, <.05).

**Table 3.** Global Moran's I for Residuals of two-Level Logistic Regression Models on Adherence to Clinical Recommendations Across 24 Health Care Services at the MS Level in Switzerland.

**Group**	Health Care Service	Global Moran’s I at MS level	Effect
**Screening**	Colon cancer screening	0.08	
	Breast cancer screening	0.07	
	Prostate cancer screening	0.51***	Strong
	Osteoporosis screening	0.10*	Small
**Diagnosis**	DM: HbA1c test	0.25***	Medium
	DM: kidney exam	0.19**	Small
	DM: LDL test	0.24***	Small
	DM: eye examination	0.12*	Small
	TSH screening	0.29***	Medium
	POCR	0.07	
**Primary prevention**	Influenza vaccination	0.01	
**Secondary prevention**	AMI: aspirin	0.06	
	AMI: statin	0.16**	Small
	AMI: beta-blocker	0.09.	
	AMI: ACE/ARB	0.01	
	AMI: P2Y12 inhibitors	0.01	
	PPI with NSAID	0.19**	Small
	PAD: statin	0.03	
	Afib: anticoagulation	0.09.	
	GCC with new DMARD	−0.01	
**Treatment**	Benzodiazepines	0.27***	Medium
	Proton pump inhibitors	0.08.	
	Outpatient procedures	0.13*	Small
	Caesarean section	0.07	

Significance values: ***<0.001, **<0.01, *<0.05, .<0.1. We considered Moran’s I (MI) values of > = 0.1 as small, of > = 0.25 medium, and > = 0.5 as strong effects.

**Figure 2.**
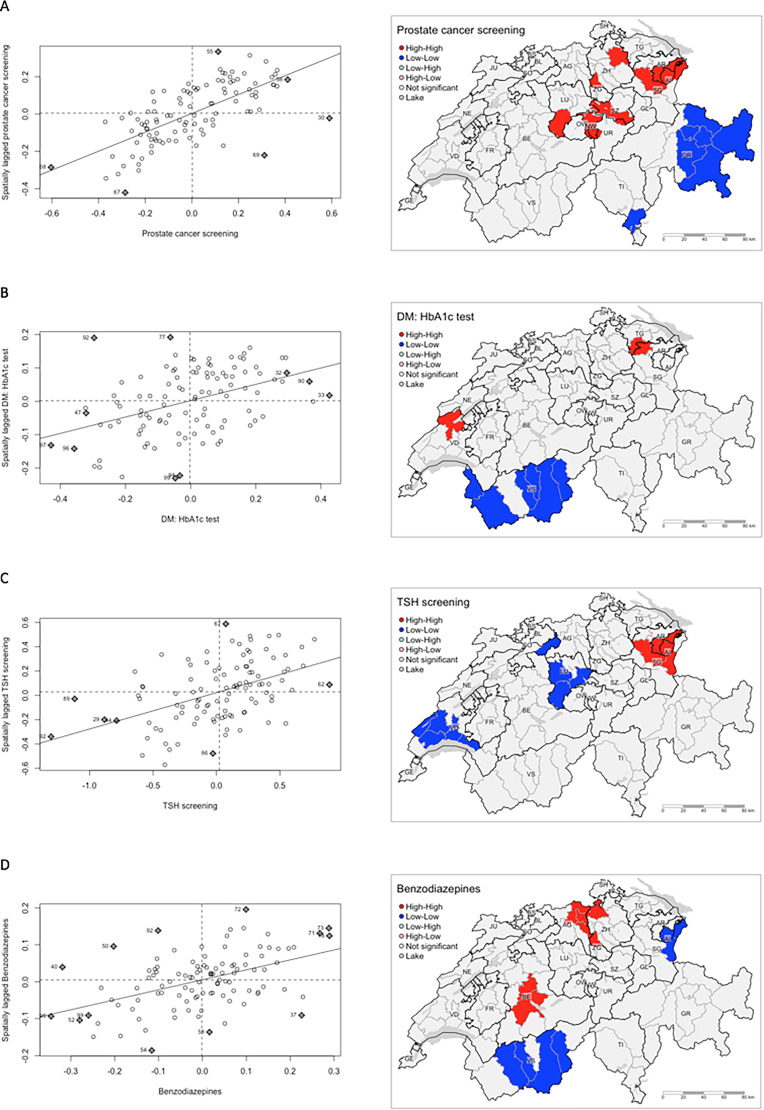
Moran scatterplots (left) and local indicators of spatial association (LISA) maps (right) for adherence patterns across A) prostate cancer screening, B) diabetes testing with HBA1c, C) TSH screening, and D) Benzodiazepines as types representing health care services in Switzerland.

We only considered adherence to those services with statistically significant MI values of > = .25 and > = .50 exhibiting medium and strong global spatial autocorrelation (clustering), respectively, for further identification of local spatial associations (clusters) (Figure 2). For all others, refer to the supplementary file, Figure S5. In general, no overall systematic spatial patterns could be identified across the services. Adherence to clinical recommendations for four services showed medium and strong global spatial clustering and hence also exhibited local spatial clusters: Prostate cancer screening, DM: HbA1c test, TSH screening, and benzodiazepines, with local clusters of above and below average adherence were differently distributed across the country and within cantons.

High values of model residuals for prostate cancer screening adherence clustered in the centre and north east parts of the country, that is, in seven cantons: Appenzell Ausserrhoden, Appenzell Innerrhoden, Luzern, Nidwalden, Schwyz, St. Gallen, and Zurich (Figure 2A, regions shaded in red). For DM: HbA1c test, high model residuals (adherence hotspots) clustered in MS regions bordering Thurgau and St. Gallen and in a northern MS region of Vaud (Figure 2B). In contrast, low model residuals (adherence cold spots) clustered across most MS regions in Valais. Adherence hotspots for TSH screening were found in MS regions across the cantons of St. Gallen, Appenzell Ausserrhoden, and Appenzell Innerrhoden, while low model residuals (poor adherence) for TSH clustered in MS regions of Luzern, North of Bern, south of Vaud (Figure 2C). For benzodiazepines, hotspots were found in central area of canton Bern and northern MS regions of canton Aargau spanning to MS regions within the canton of Zurich. Cold spots for benzodiazepines were found in neighbouring MS regions of Appenzell Innerrhoden and St. Gallen, and in Valais (Figure 2D).

### Qualitative Assessment of Possible Influencing Factors for Small-Area Variation

The variation patterns illustrated in the maps shown in Figure 2 were critically reviewed on the basis of our postulated framework (Table 4). Indeed, the maps showed examples of patterns that were compatible with possible political influences (type 1) or cross-cantonal care network effects (type 2). Examples for type 1 patterns include prostate cancer screening, where large parts of the canton of Graubünden were identified as cold spots. A second example was HbA1hc screening in the canton of Valais, which was found to be less frequently executed. For TSH screening, connected cold spots were found for the cantons of Vaud and Lucerne, which covered more than half of the cantonal areas.

**Table 4.** Characteristics of Local Clusters at the MS Level with Respect to the Cantonal Boundary and Remoteness. H-H: Above Average Values of MS-Level Residuals in Neighbouring Regions and Vice Versa (LL).

Group	Health Care Service	Type 1: Cantons with individual local clusters (count, type)	Type 2: Cantons with local clusters spanning across cantonal boundaries (type)	Type 3: Cantons with clusters in remote areas
**Screening**	Prostate cancer screening	Graubünden (1, LL), Lucerne (1, HH), Ticino (1, LL) Zurich (2, HH)	Nidwalden, Obwalden, Zug (HH), Appenzell-Ausserrhoden, Appenzell-Innerrhoden, Sankt Gallen (HH)	/
**Diagnosis**	DM: HbA1c test	Vaud (1, HH), Valais (2, LL)	Sankt Gallen, Thurgau, (HH)	/
	TSH screening	Lucerne (1, LL), Solothurn (1, LL), Vaud (1, LL)	Appenzell-Ausserrhoden, Appenzell-Innerrhoden, Sankt Gallen (HH)	
**Treatment**	Benzodiazepines	Berne (1, HH), Valais (1, LL)	Aargau, Zurich, Zug (HH) Appenzell-Innerrhoden, Sankt Gallen (LL)	/

Examples for type 2 variation (possible cross-cantonal care networks) were found twice for the same area. TSH testing and prostate cancer screening for the area of St. Gallen and both cantons of Appenzell. The cantonal hospital in St. Gallen is one of the largest cantonal hospitals in Switzerland and an important regional centre. Therefore, a network effect is possible.

However, our qualitative assessment also showed that type 3 drivers are very hard to identify in geographically small countries such as Switzerland. There were no hot spots or cold spots that we could link to topographical idiosyncrasies with good confidence.

## Discussion

We found that in despite of controlling for potential influencing factors (eg, socio-demographic, clinical, and health insurance-related characteristics), adherence to clinical recommendations relating to 24 diverse healthcare services varied geographically across MS regions in Switzerland, also within cantons. Medium to strong statistically significant spatial patterning (ie, clustering) of adherence across MS regions was found for prostate cancer screening, DM: HbA1c test, TSH screening, and benzodiazepines. The spatially dependent adherence patterns in the use of these health care services exhibited statistically significant local clusters of above and below average adherence, which were heterogeneously distributed across MS regions, albeit with no clear overall pattern across the health care services. No single region stood out as having consistently lower or higher guideline adherence, thus suggesting that high or low adherence is not an inherent, static attribute of certain regions, but highly dependent on the type of intervention.

Regression residuals generally indicate unexplained variance in modelled estimates. For the context of clinical recommendations, upper quartile (highest 25% of the residual distribution) indicated better than expected adherence in a MS region, while the lower quartile (lowest 25%) indicated worse than expected adherence, given the adjusted individual and MS level influencing factors.^6^ However, it is important to note that some health care services (eg, prostate cancer screening, benzodiazepines) are *discouraged* in clinical recommendations. In consequence, for those services, regions in the upper quartile indicated *less* utilization while regions in the lower quartile indicated *more* utilization of a health care service that is not recommended without personalized decision-making.

### Geographic Variation of Adherence to Clinical Recommendations

Adherence to clinical recommendations varies in relation to a number of factors, including patient and provider preferences.^6^ Adherence is also influenced at various levels (from individual to provider and ecological levels), and considering that these factors vary across geographic space, it is not surprising that our data also exhibited spatial heterogeneity in adherence to clinical recommendations, when visualized in geographic maps. However, our study was one of the first to investigate adherence to clinical recommendations across multiple health care services (N = 24), based on adjusted residuals of multilevel models of health care utilization constructed from health insurance claims data, as detailed elsewhere.^6^ The identified spatial heterogeneity is of note as it indicates spatial variation of extreme values (highest and lowest 25% of the distribution) across MS regions in Switzerland and across all 24 health care services. As such, we identified unwarranted variation in health care usage in all or most regions within a political unit (canton).

### Spatial Clustering of Adherence

We systematically assessed the geographic variation of adherence to clinical recommendations and detected small to strong spatial clustering in about half (11 out of 24) of the investigated health care services. By investigating the medium and strong clustering further in local spatial cluster analysis, we found that both better and worse than expected adherence to four services exhibited spatial dependence across MS regions, with similar (above or below average) adherence concentrating in regions that were also geographically close to each other. This finding lends itself to at least two possible interpretations. *First,* we provide novel evidence that among a large set of health care services, neither systematic nor common geographic patterns could be found. *Second,* although we had previously adjusted for influencing factors at the individual person level such as higher annual deductible level, supplementary insurance, or having chosen a managed care model,^6^ adherence to clinical recommendations continues to vary geographically, depending on the health care service and the respective clinical recommendation for its usage. Furthermore, we noted that only four out of the 11 services exhibited at least medium or strong global spatial clustering that we considered for further spatial analysis.

### Hotspots and Cold Spots of Adherence

Four health services also showed statistically significant local spatial clusters of adherence across MS regions. These clusters were differently distributed across geographic space, depending on the health care service and the respective clinical recommendation. We noted hotspots of increased adherence and also cold spots of decreased adherence. These hot and cold spots were found at the MS level and sometimes spread across cantonal borders. For example, we found adherence hotspots for prostate cancer screening in seven cantons and cold spots were found in two cantons. Prostate cancer population-based screening is debated and not recommended by the clinical guidelines. Therefore, the identified adherence cold spots indicate sub-cantonal regions in which the utilization of the service was particularly pronounced, although its usage was discouraged by clinical recommendations (at least in the absence of shared decision-making). While similar programs supporting or discouraging a specific health service may be implemented across various cantons, adherence may largely differ, eg, driven by varying implementation and communication strategies, making specific assumptions about possible associations between influencing factors and recommendation adherence difficult. Nevertheless, we have at least three generic explanations for the hotspots and cold spots identified. *First*, since regional clusters were found at the sub-cantonal MS level, we assume that there are locally specific factors associated with health care utilization and adherence to clinical recommendations that go beyond our initial regression analyses. For example, these locally specific factors may be described as place-based social determinants of health (SDH), which have been defined as the circumstances in which people are born, grow, live, work and age, such as education, socioeconomic status, social support networks, neighbourhood socio-ecological environments, and health care access.^11–13^ In this context, the density of available services may differ across MS regions with higher densities of a respective service in some regions versus lower densities of services in other regions leading to more or less utilization of the service provided in the areas, respectively. Furthermore, the utilization of a health care service such as prostate cancer screening most often follows a decision process between the patient and the provider. Various influencing factors may play a role in this decision process, such as the patient or provider’s preferences, age, or professional experience guiding the shared decision about the service. Low density of service providers in these areas combined with an attitude that is in favour or against this service (irrespective of its clinical recommendation) may have further contributed to local patterns (hot or cold spots) of adherence. *Second*, place-based SDH may be particularly important across neighbouring MS regions, sometimes even across canton boundaries. For example, similar health care needs in the population (eg, demographic structure), physician density (eg, availability of health services in the region), or local transit options (eg, accessibility of health services through public transport) may have contributed to the hot or cold spots of adherence to clinical recommendations. Further studies need to test the functional relationship between the geographic variation of access (eg, accessibility and availability) and the health care need in the population. *Third*, we may have identified local spatial spill-over effects, that is, influencing factors within one region may have affected health care utilization of neighbouring regions, although these factors may not particularly be important within the neighbouring regions themselves. For example, when physician density (combined with a specific attitude towards or against a specific recommendation) in one MS region is higher than in the neighbouring MS regions, people may utilize the service provided in the one region where the density is higher, sometimes even across cantonal borders.

Our findings were consistent with a type 1 pattern, that is, clusters predominantly fell within a political (cantonal) boundary, which may indicate an influence of local health policy and guidelines. Still, other influence factors cannot be excluded, such as health care access, patient socio-demographics not covered by our data source, or healthcare system-related factors.^2,4^ Moreover, it should be noted that our framework mainly holds for adherence to recommended actions (hot spots). Cantonal policies will encourage the use or abandonment of a certain treatment. If this is found, a health policy / political effect is more likely. If a pattern shows cold spots, this is more likely a network effect (type 2). No canton will actively encourage care providers to do something that is not recommended (but influential health care providers may). In sum, these examples emphasize the utility of our joint approach of geospatial analysis and framework-based, qualitative analyses to generate further hypotheses about drivers of small area geographic variation.

### Strength and Limitations

Our study has the following limitations. Although our data is from one of the largest available datasets representing persons insured by the statutory health insurance in Switzerland, it still misses a significant part of the Swiss population and may not be representative across the MS regions. Future studies should aim at combining data from various insurance companies to make the dataset as representative as possible at the MS level of analysis. Furthermore, Swiss health insurance claims data do not contain diagnosis information, patient and provider preferences, or provider characteristics (eg, years in practice), which limited our and previous studies to identify more health care services and respective adherence to clinical recommendations. As such, our set of 24 health care services is somewhat arbitrarily defined, although the selection of these services was based on a comprehensive and systematic approach detailed elsewhere.^6^ Future studies should investigate the geographic variation of adherence with other data for comparisons across time.

## Conclusions

Notwithstanding the above-mentioned limitations, this is the first study systematically to identify geographic variation in the adherence to clinical recommendations in Switzerland. Although there was no overall spatial pattern in adherence to clinical recommendations across the 24 health care services considered, we identified health care services that exhibited statistically significant spatial dependence in adherence. For these, we provided evidence about the locations of local clusters, that is, we identified MS regions that exhibited better (hotspots) or worse than expected adherence (cold spots). In other words, some regions in Switzerland and the doctors or patients within do use specific health care services less or more than elsewhere. If we know where these pockets are, we can tackle the issues and see why that is the case, based on additional local studies. Therefore, future studies are warranted to investigate the place-based SDH responsible for the sub-cantonal variation in adherence to clinical recommendations in Switzerland and elsewhere over time.

## Supplemental Material

sj-docx-1-hme-10.1177_23333928221097741 - Supplemental material for Small Area Variation of Adherence to Clinical Recommendations: An Example from Switzerland Click here for additional data file. Supplemental material, sj-docx-1-hme-10.1177_23333928221097741 for Small Area Variation of Adherence to Clinical Recommendations: An Example from Switzerland by Oliver Gruebner, Wenjia Wei, Agne Ulyte, Viktor von Wyl, Holger Dressel, Beat Brüngger, Caroline Bähler, Eva Blozik and Matthias Schwenkglenks in Health Services Research and Managerial Epidemiology
