# Supplementary material for: Small Area Variation of Adherence to Clinical Recommendations: An Example from Switzerland
Source: Health Serv Res Manag Epidemiol. 2022 May 11;9:23333928221097741. doi: 10.1177/23333928221097741 (PMC9102215; doi:10.1177/23333928221097741)
Supplement: sj-docx-1-hme-10.1177_23333928221097741 - Supplemental material for Small Area Variation of Adherence to Clinical Recommendations: An Example from Switzerland [file sj-docx-1-hme-10.1177_23333928221097741.docx]

**Small area variation of adherence to clinical recommendations**

**An example from Switzerland**

**Supplementary file**

| 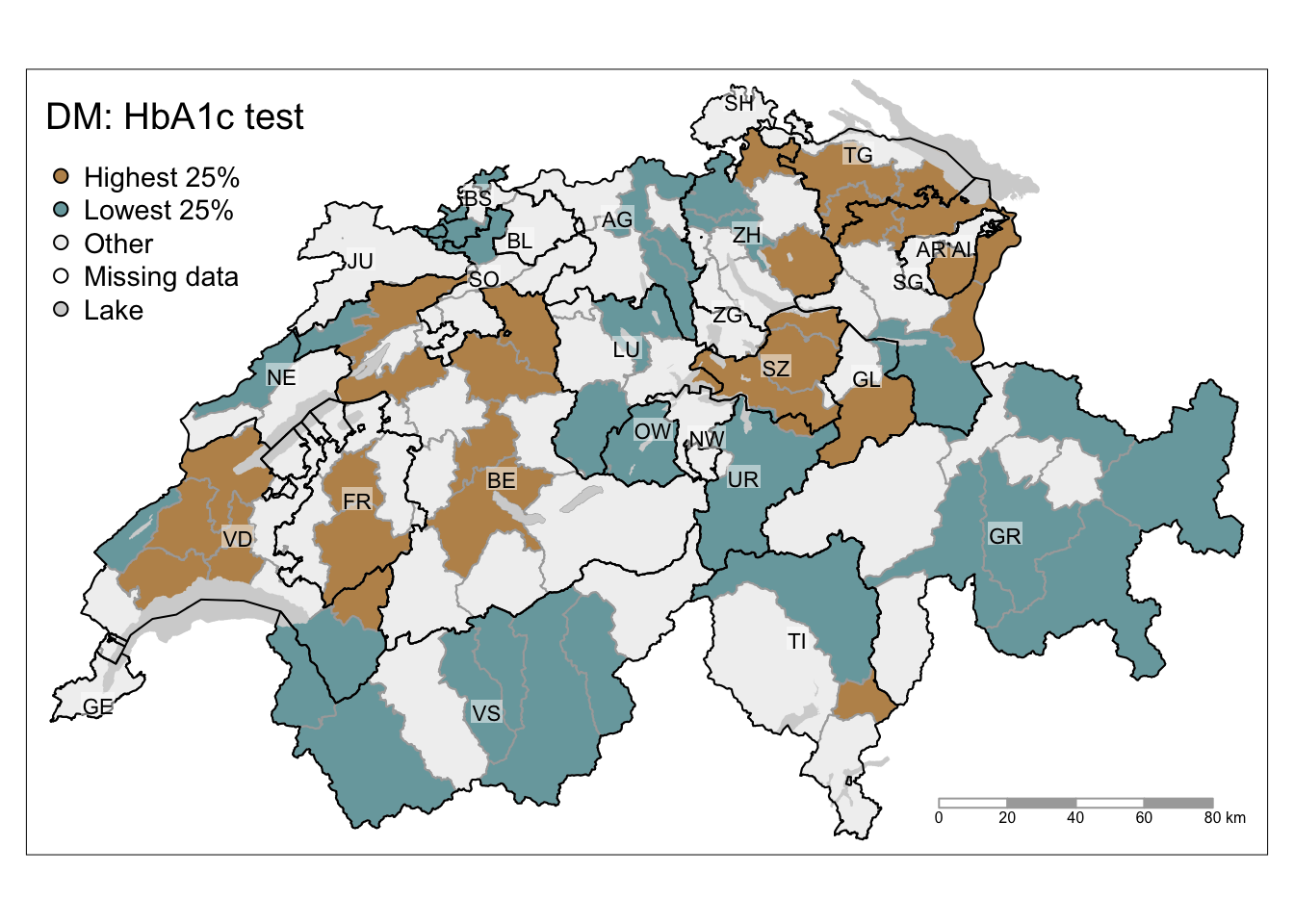 | 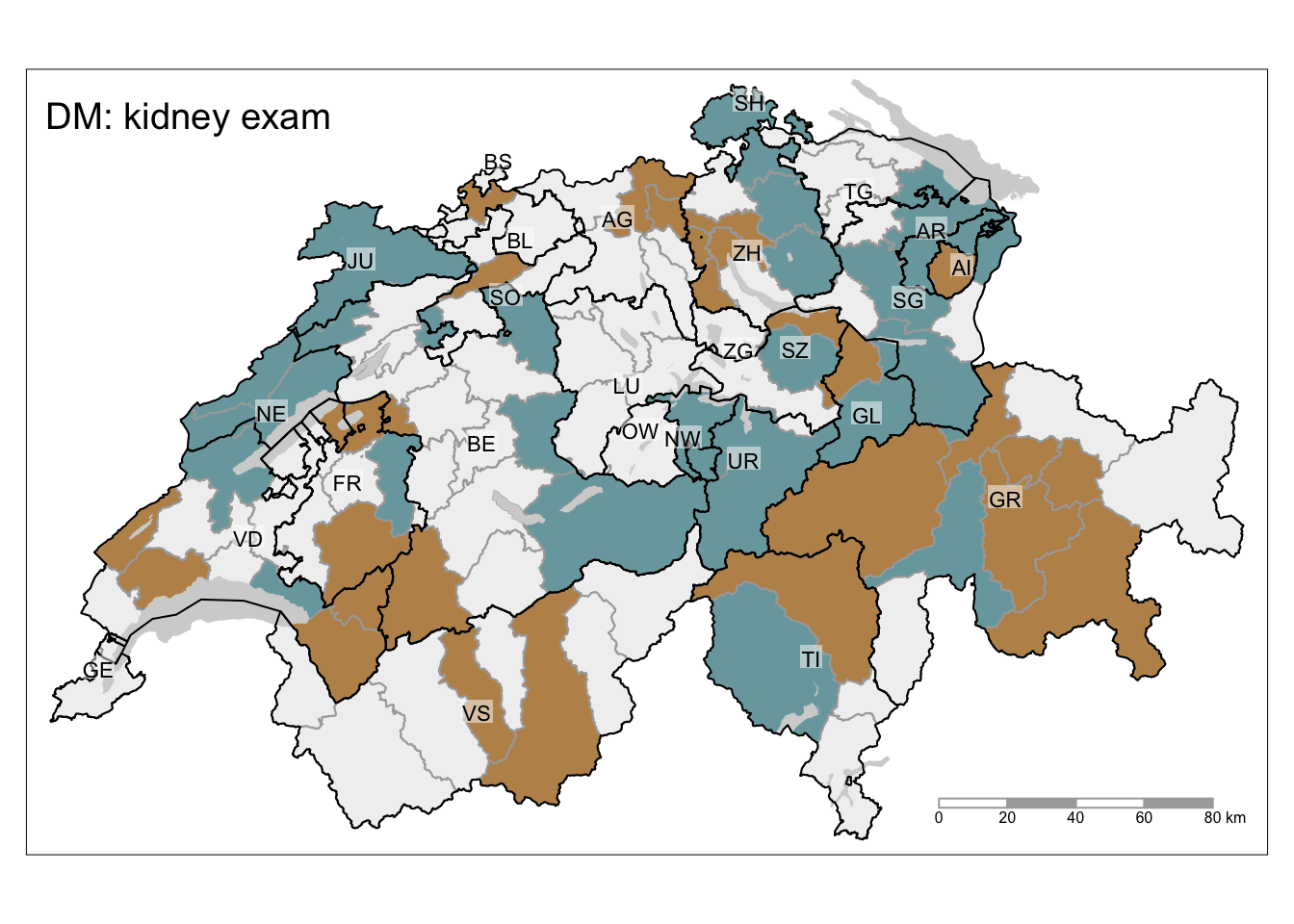 |
| --- | --- |
| 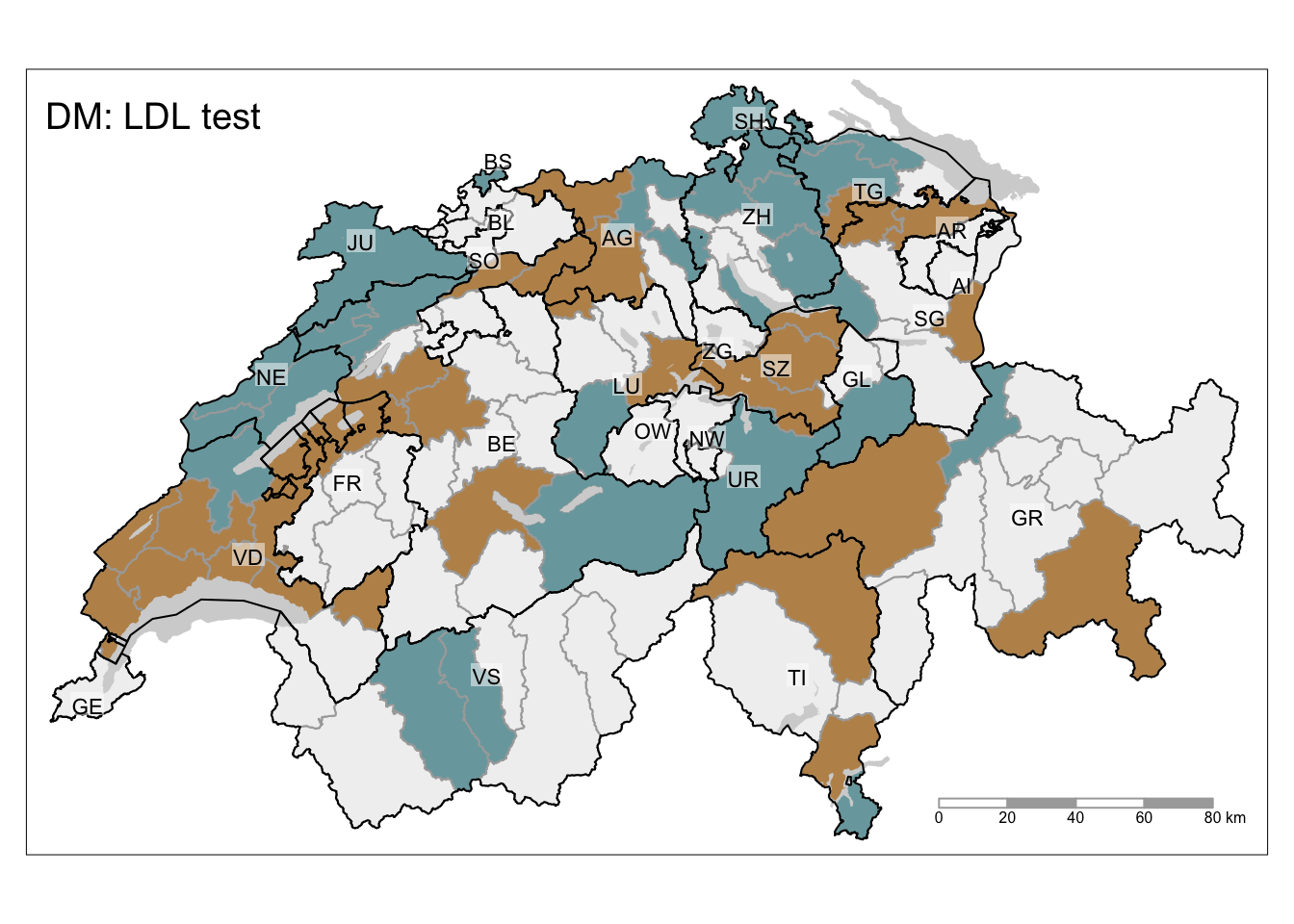 | 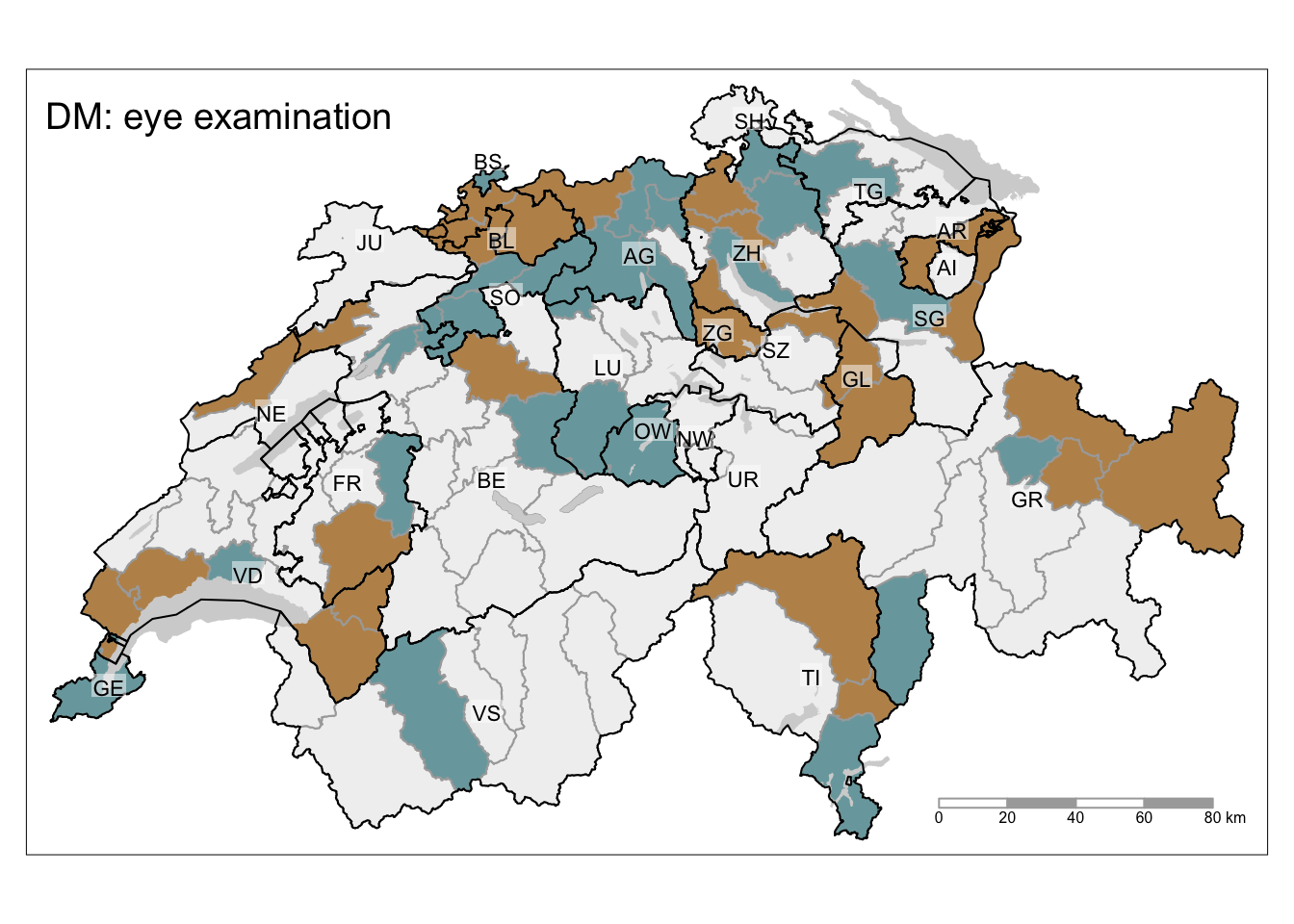 |
| 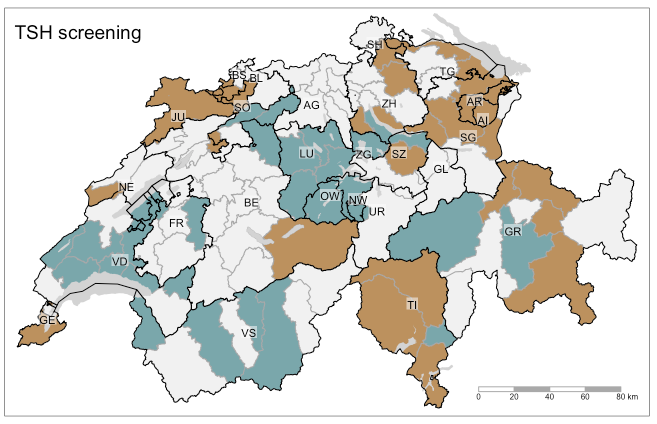 | 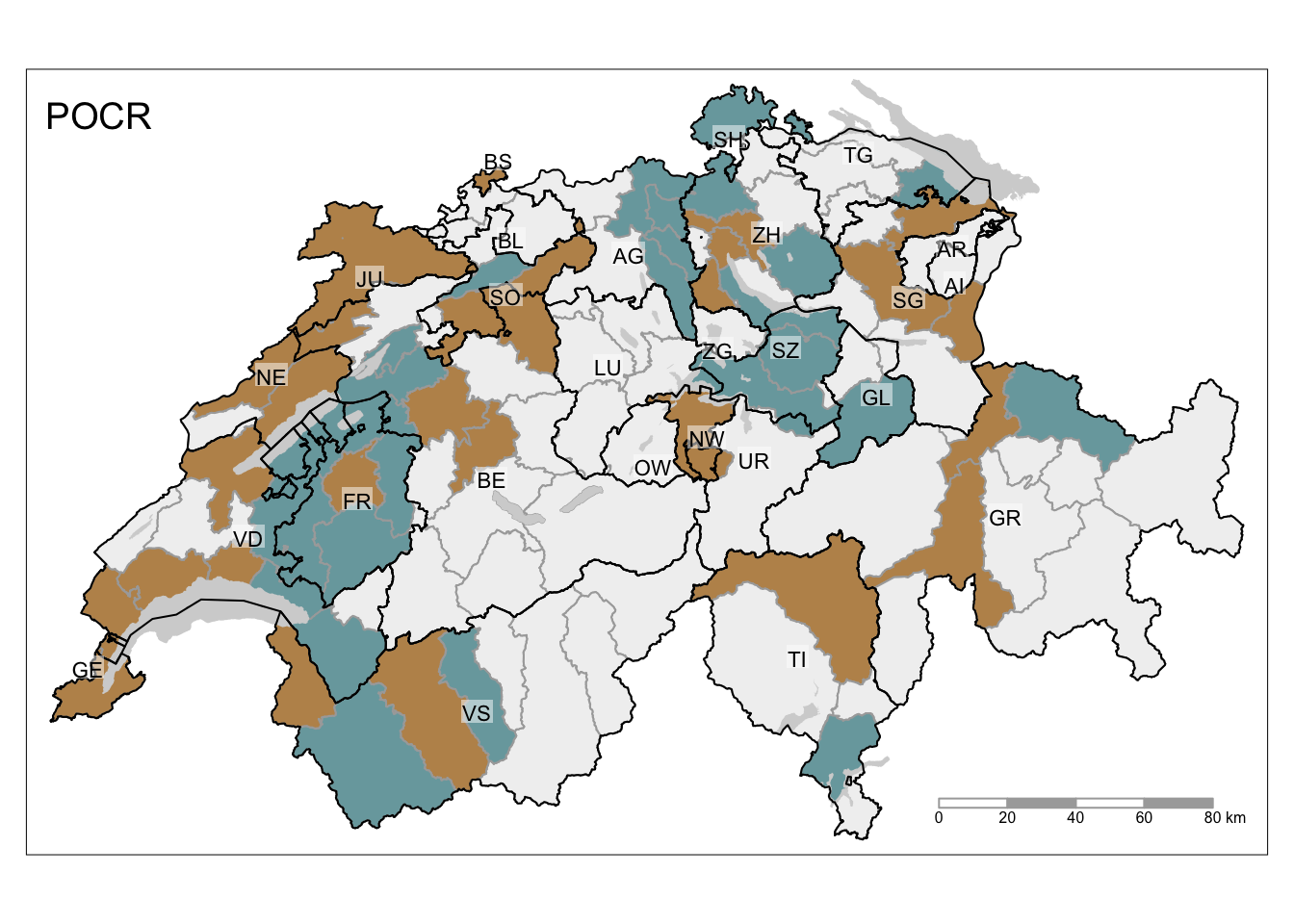 |

Figure S1: Geographic variation across the diagnosis group representing types of health care services


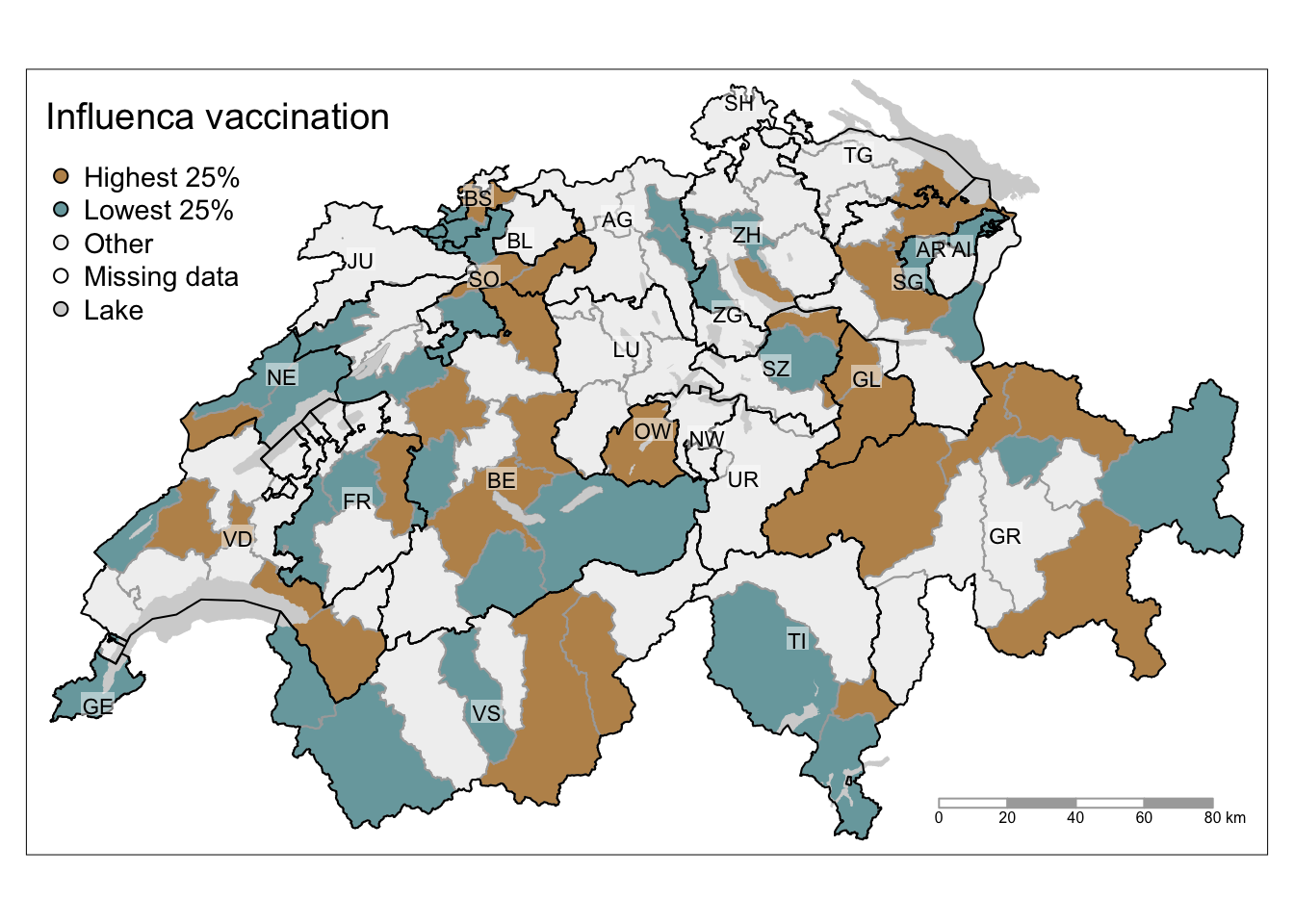


Figure S2: Geographic variation for primary prevention (influenza vaccination) representing types of health care services

| 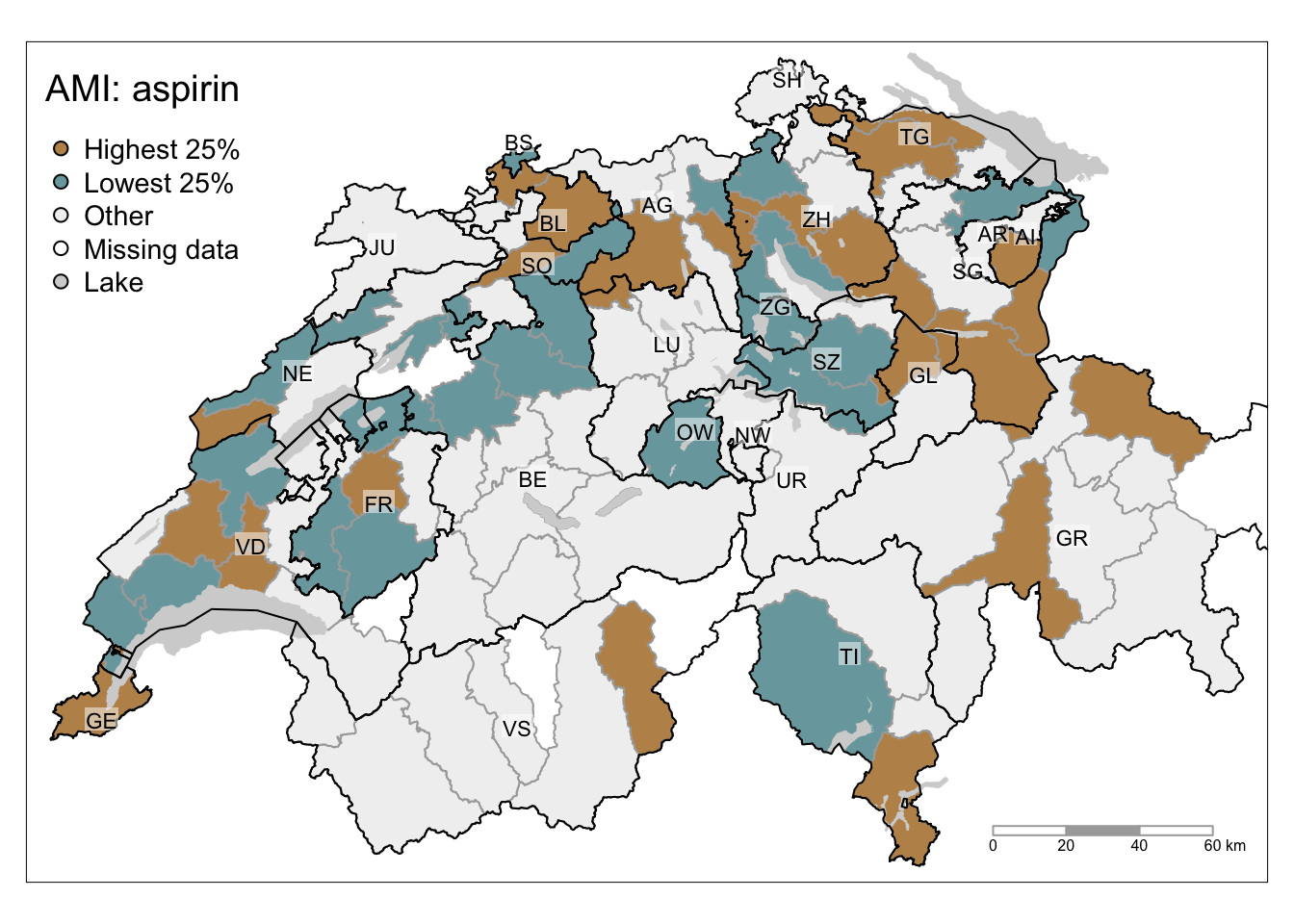 | 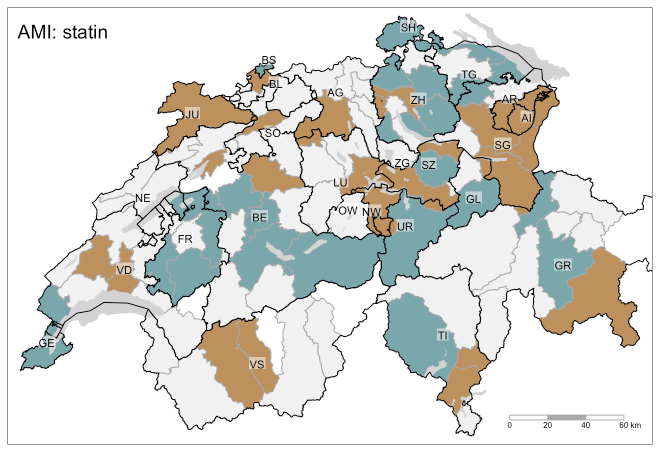 |
| --- | --- |
| 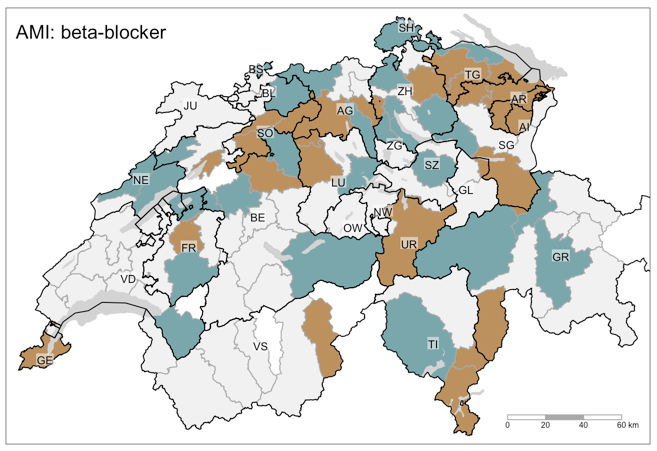 | 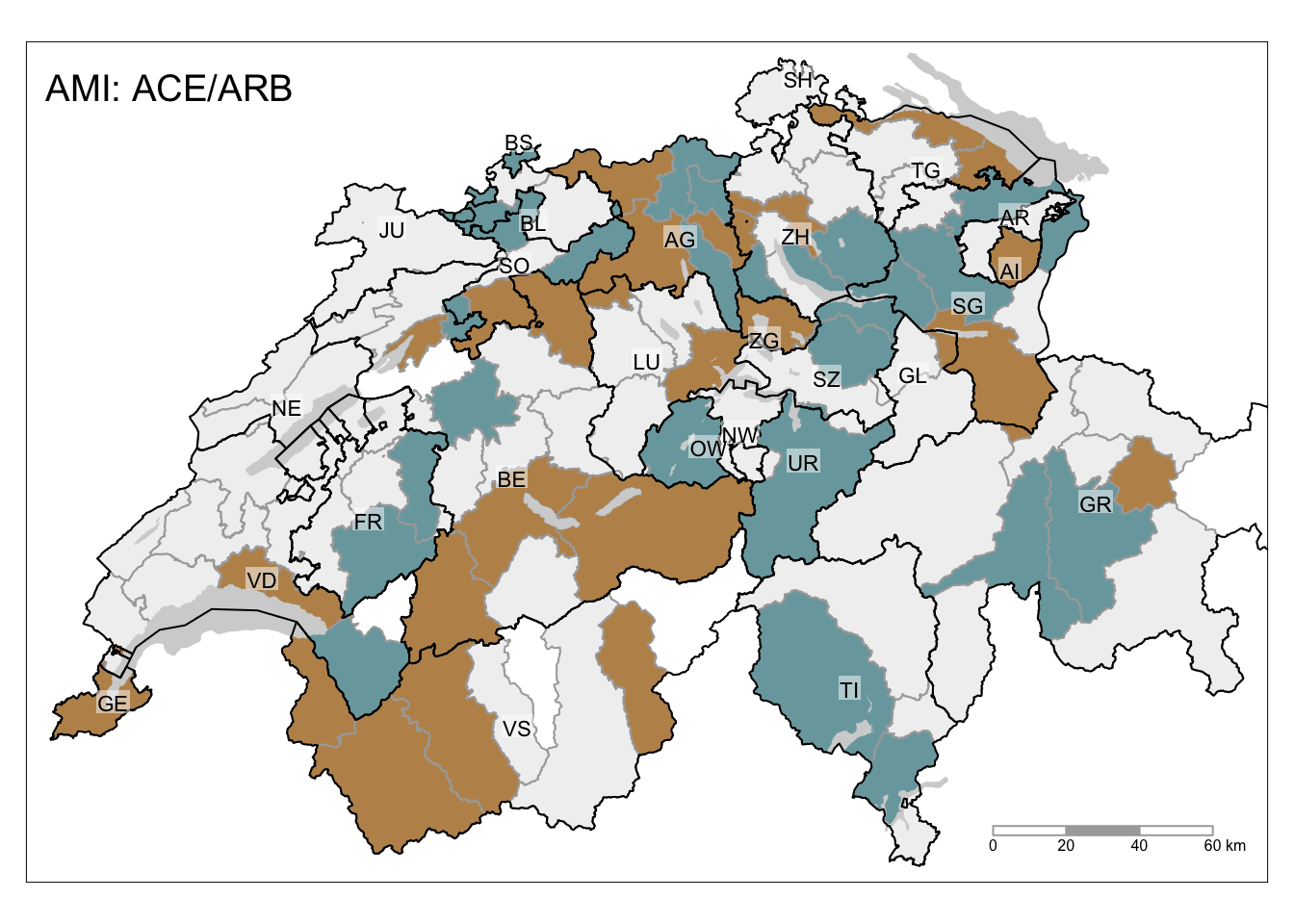 |
| 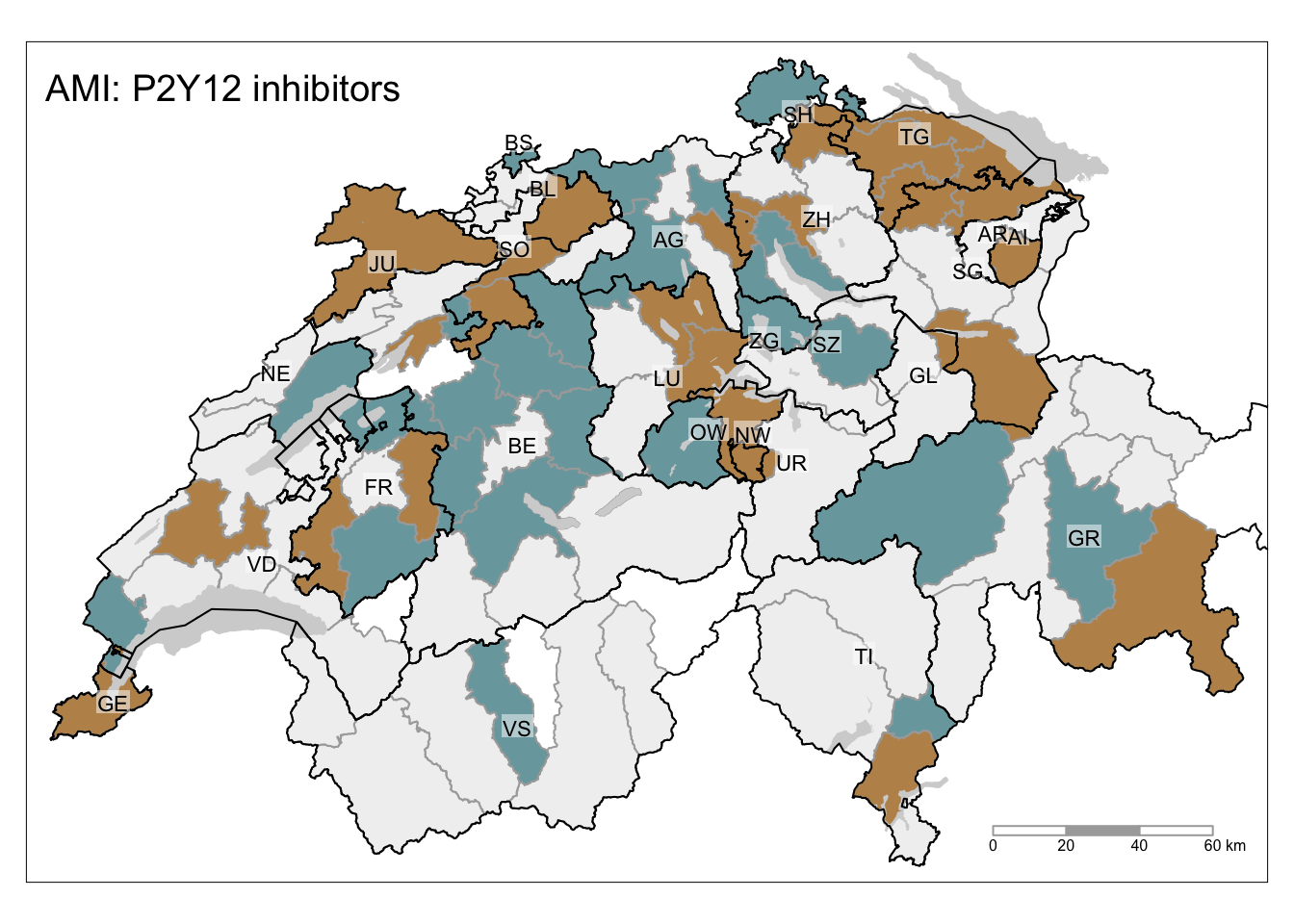 | 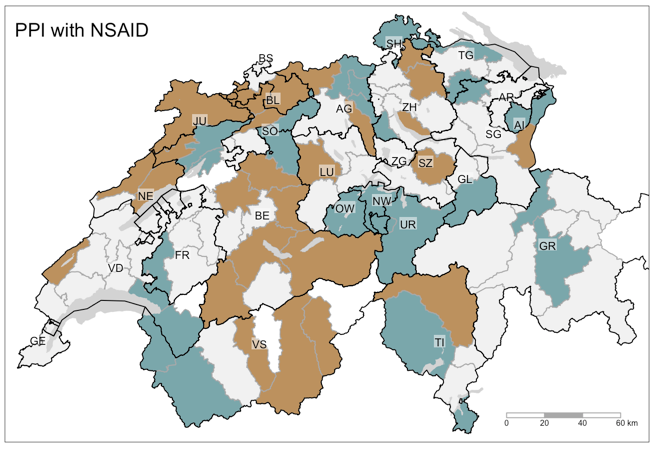 |
| 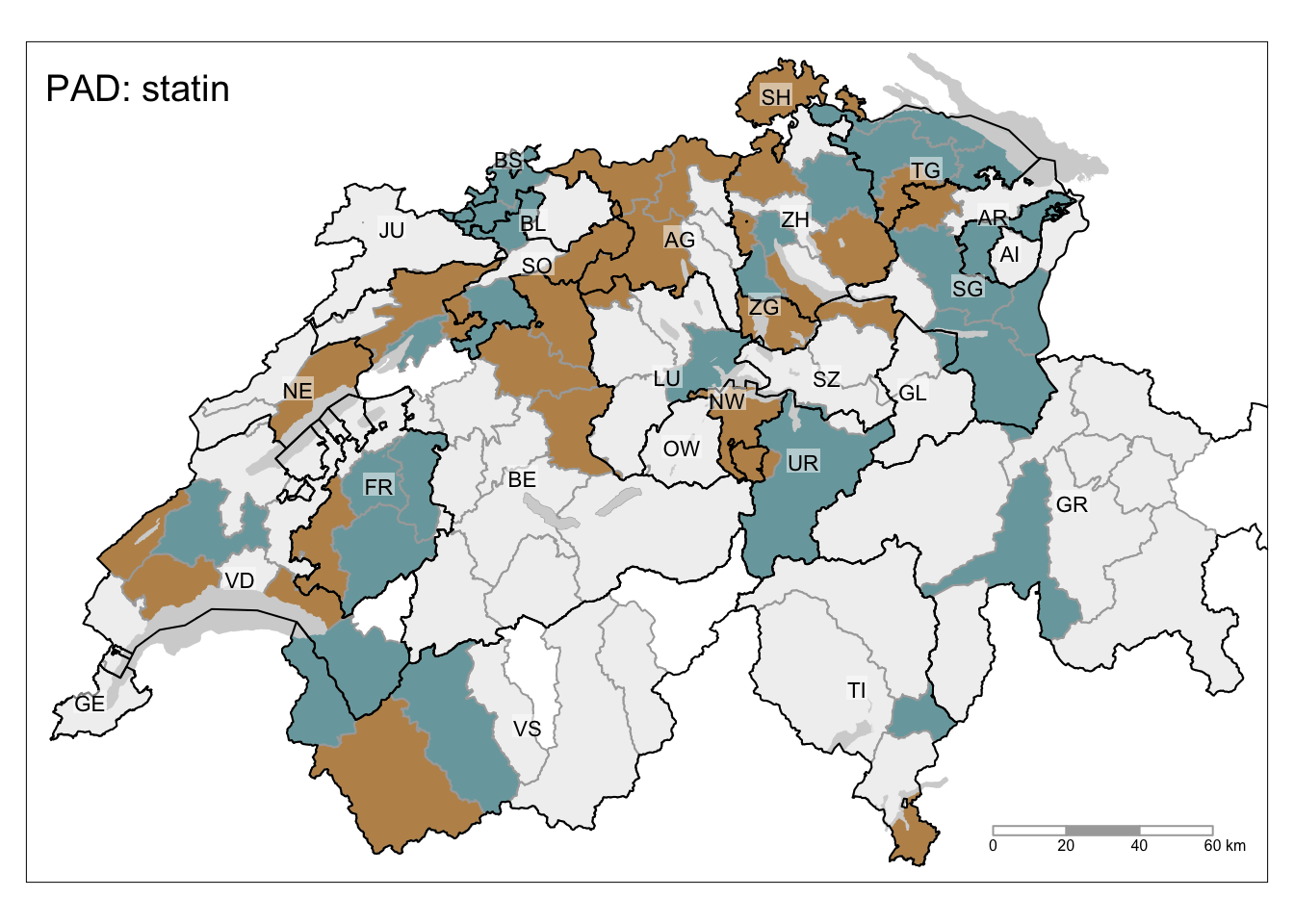 | 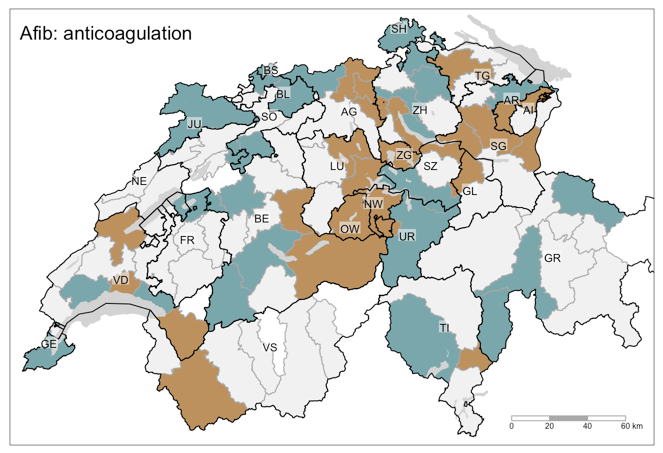 |
| 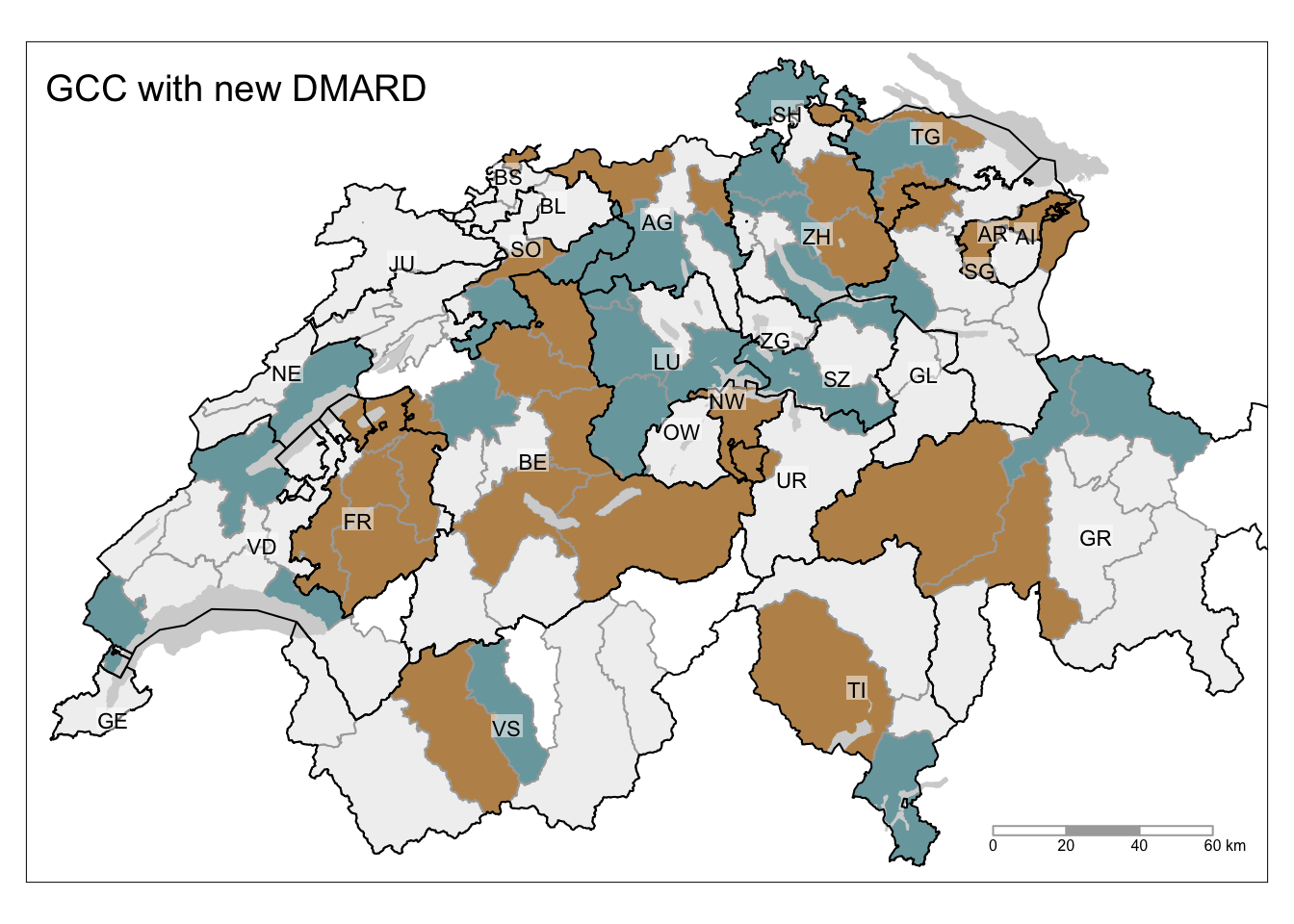 |  |

Figure S3: Geographic variation for the secondary prevention group representing types of health care services

| 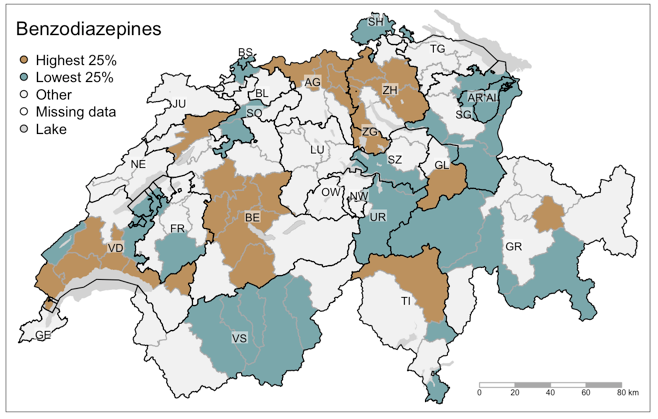 | 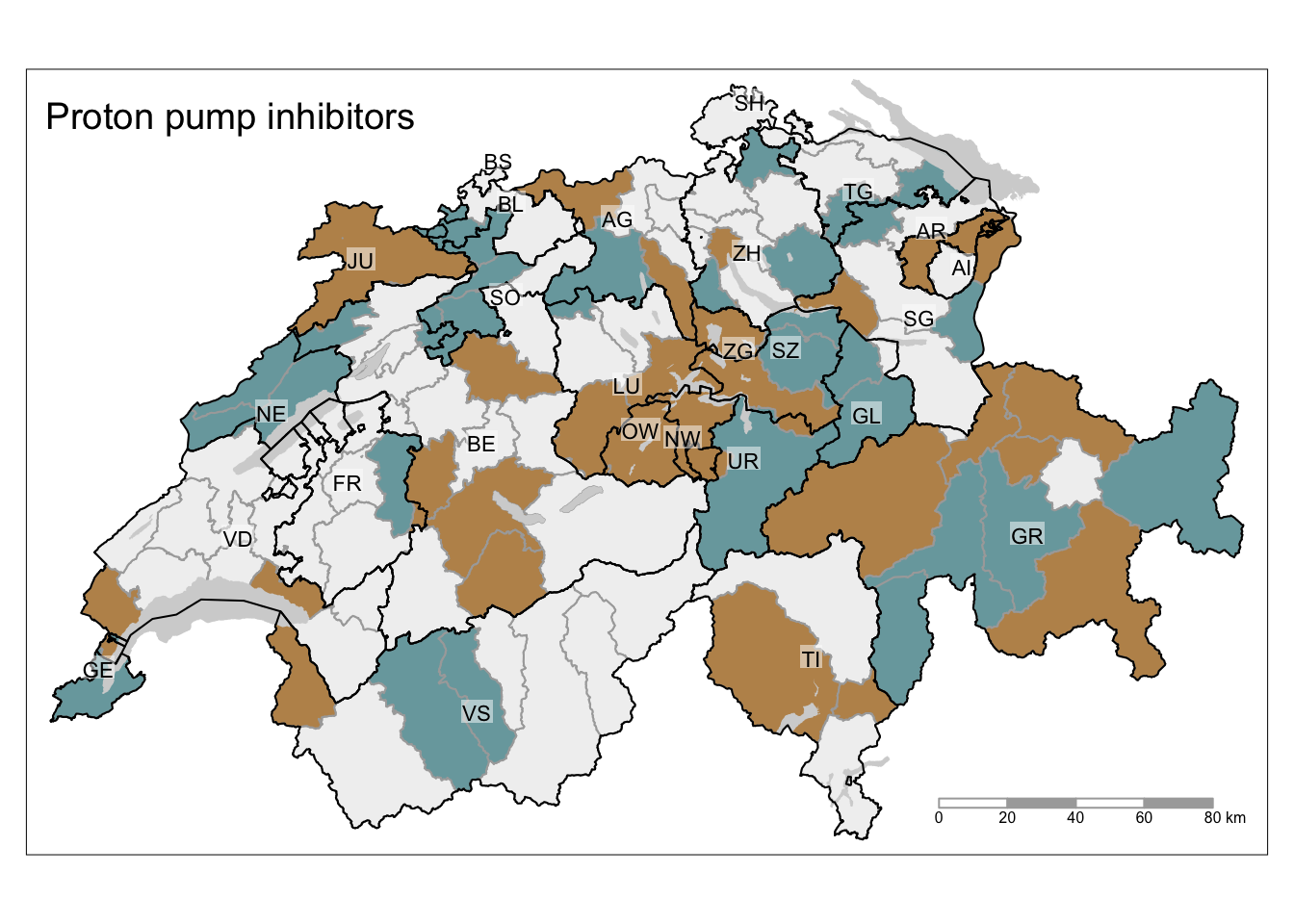 |
| --- | --- |
| 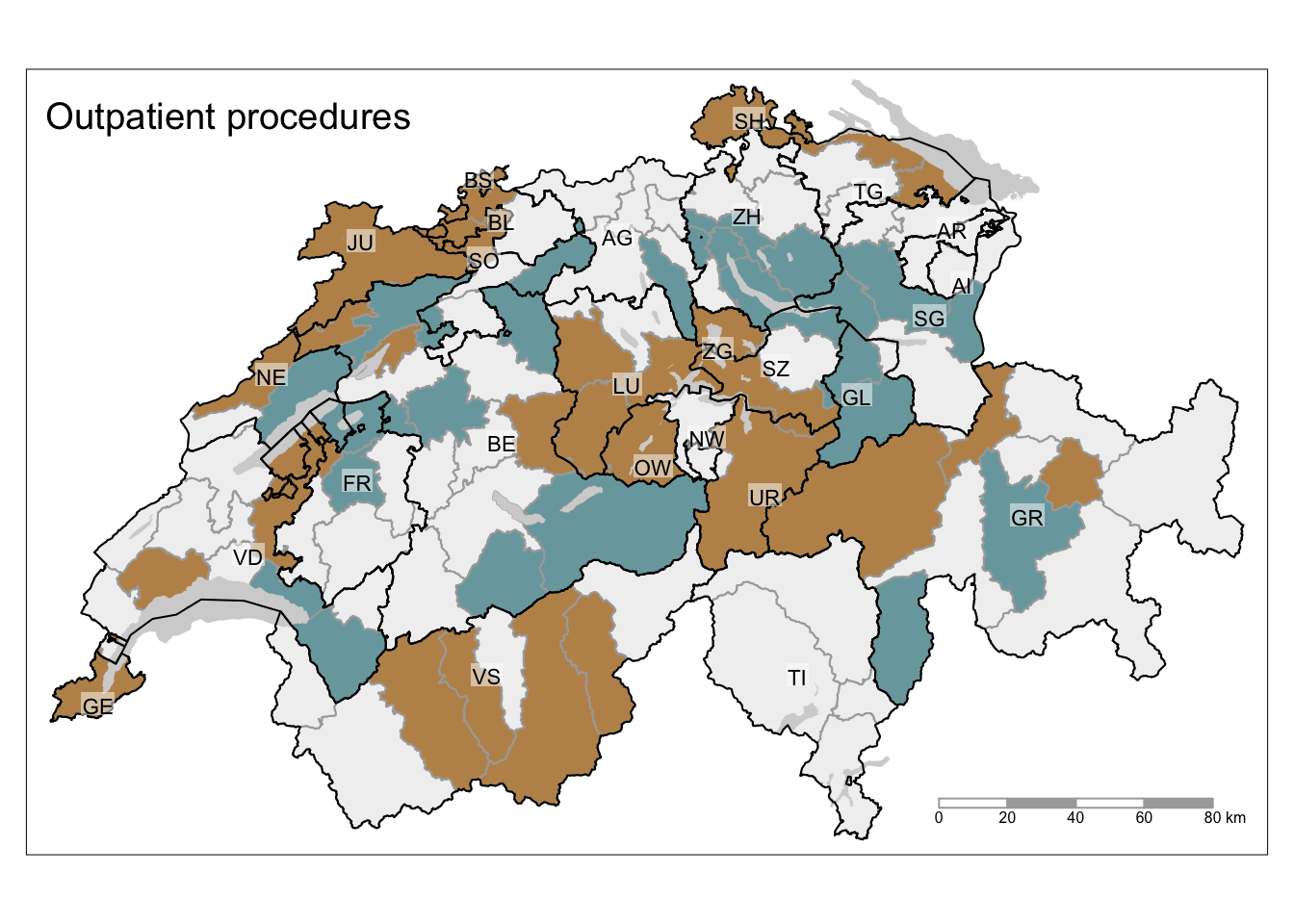 | 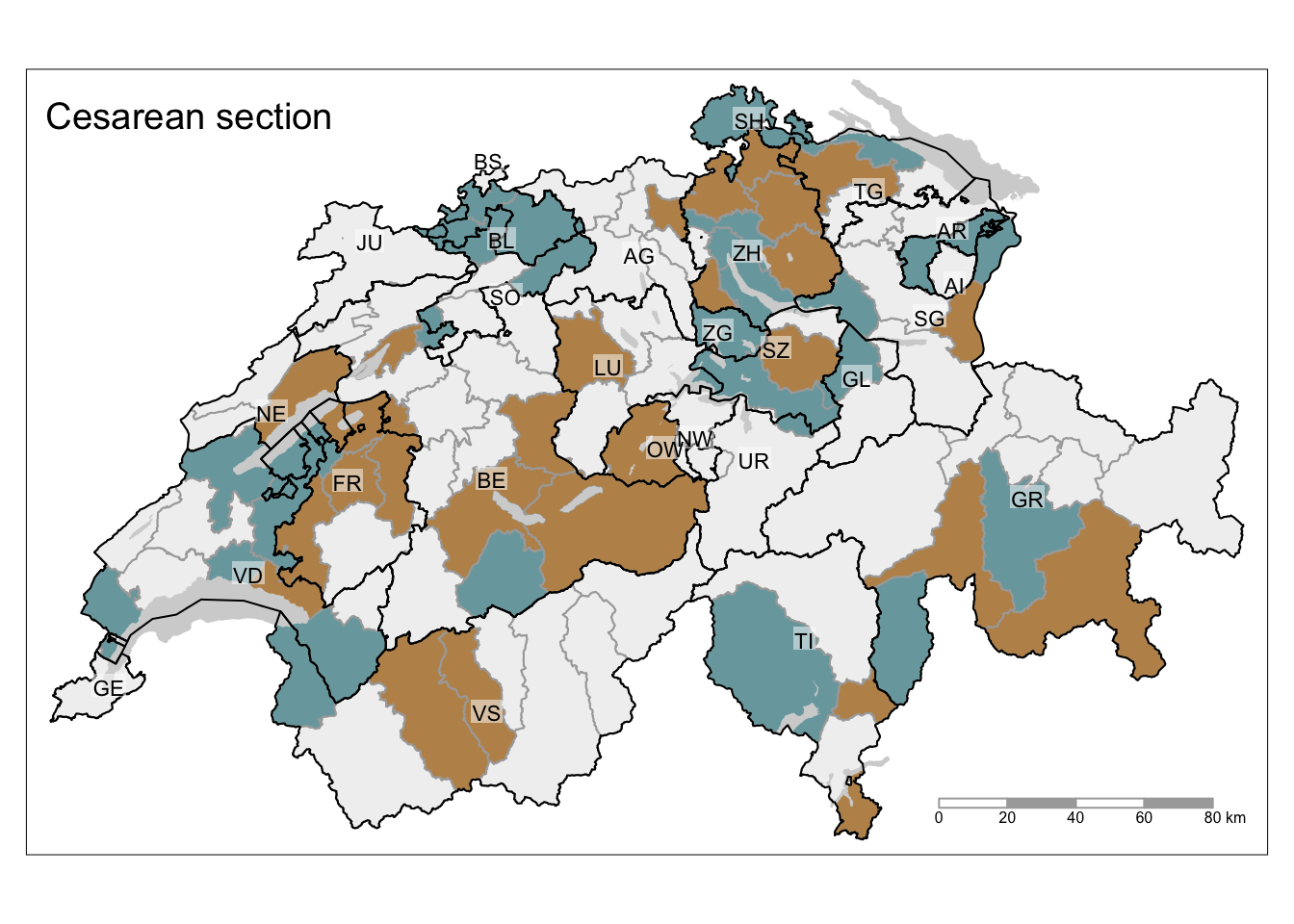 |

Figure S4: Geographic variation across the treatment group representing types of health care services

| 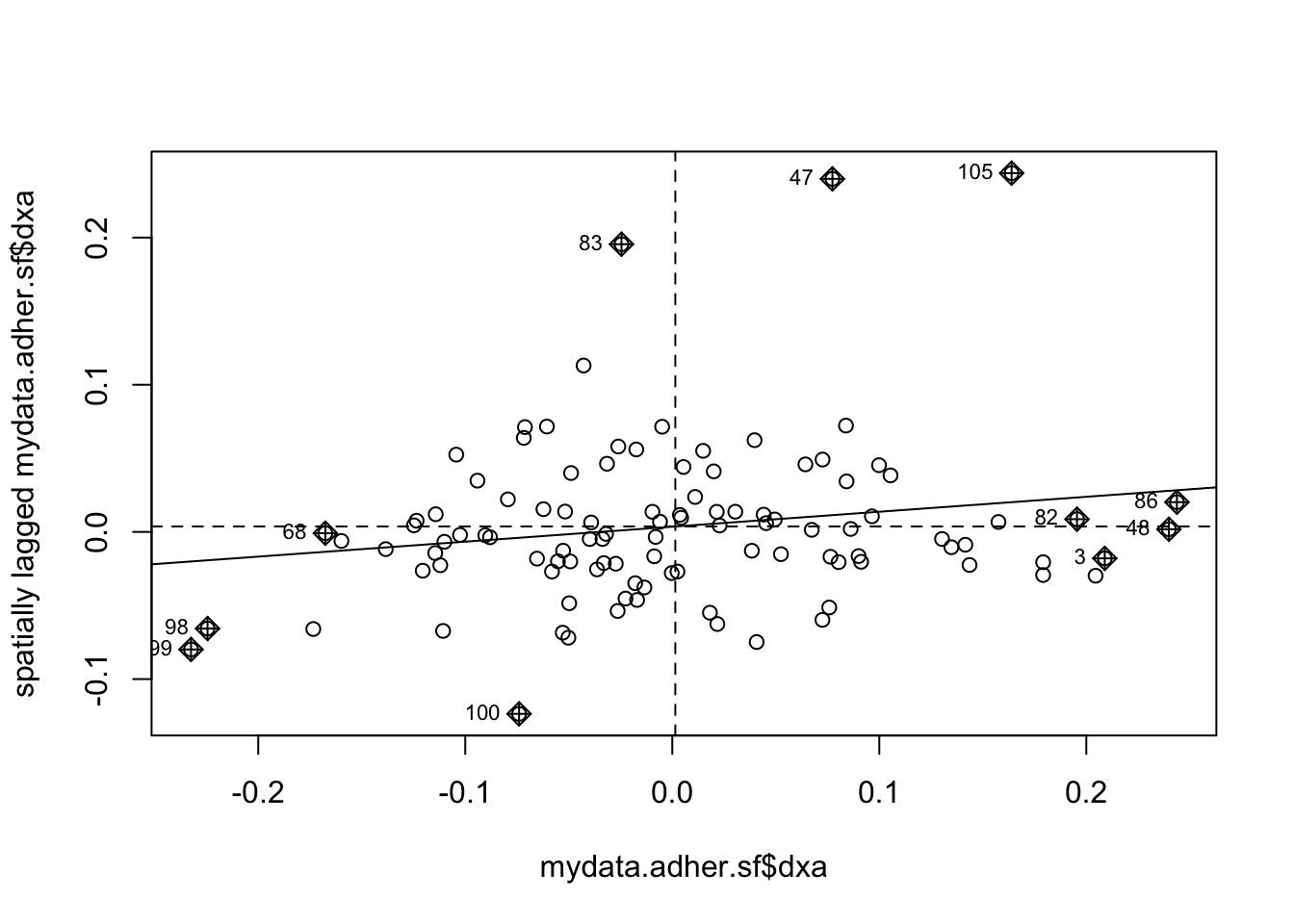 | 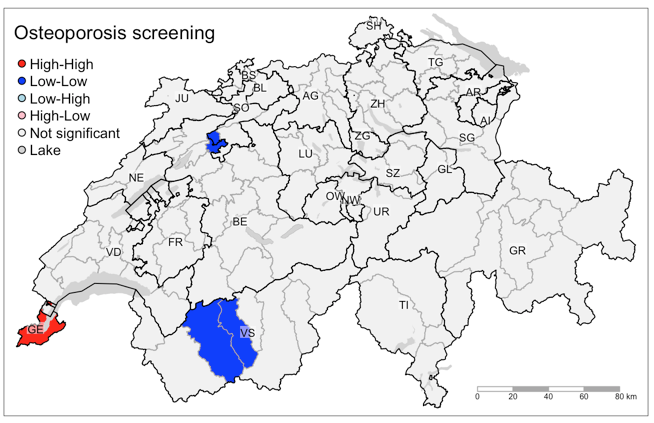 |
| --- | --- |
| 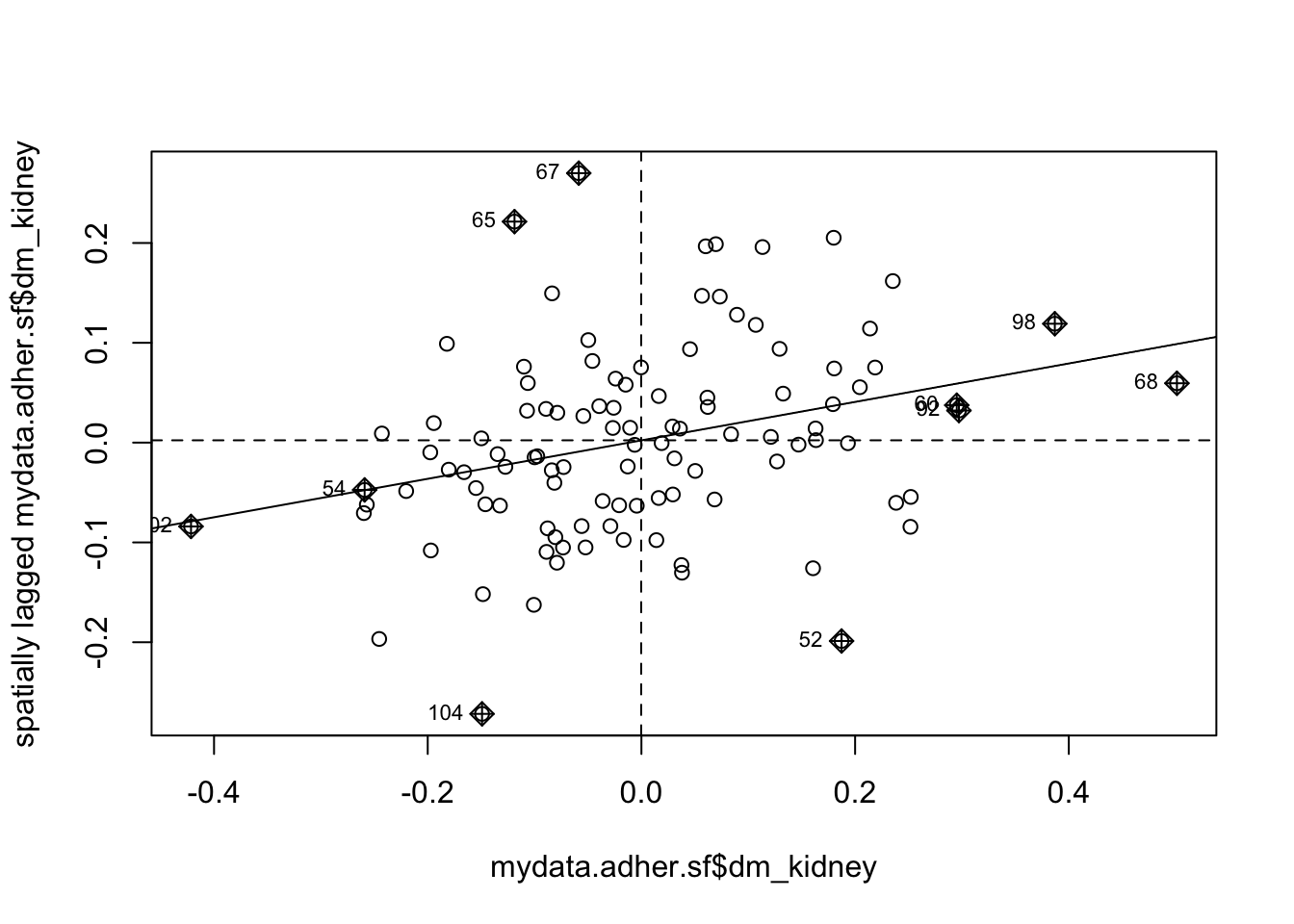 | 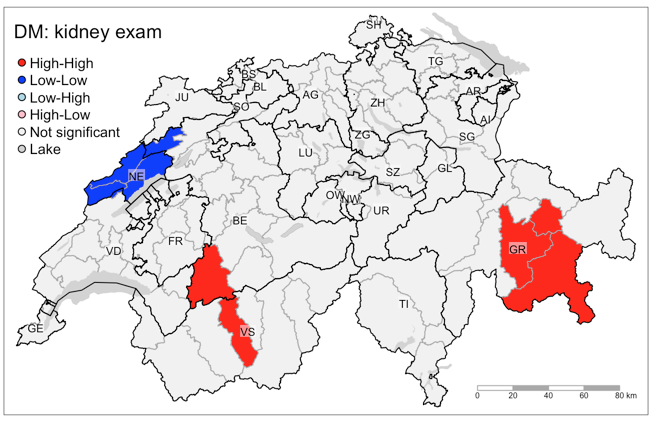 |
| 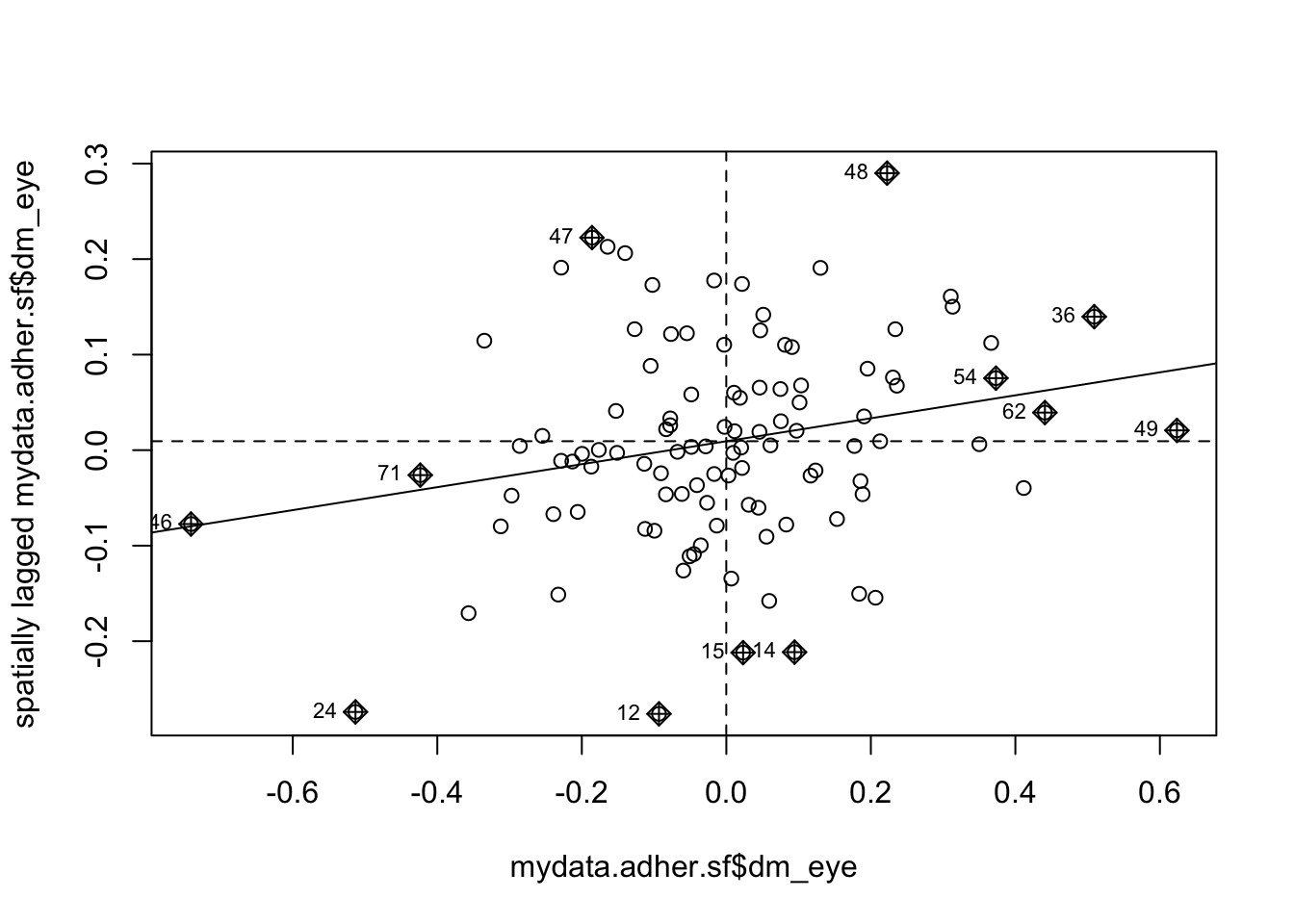 | 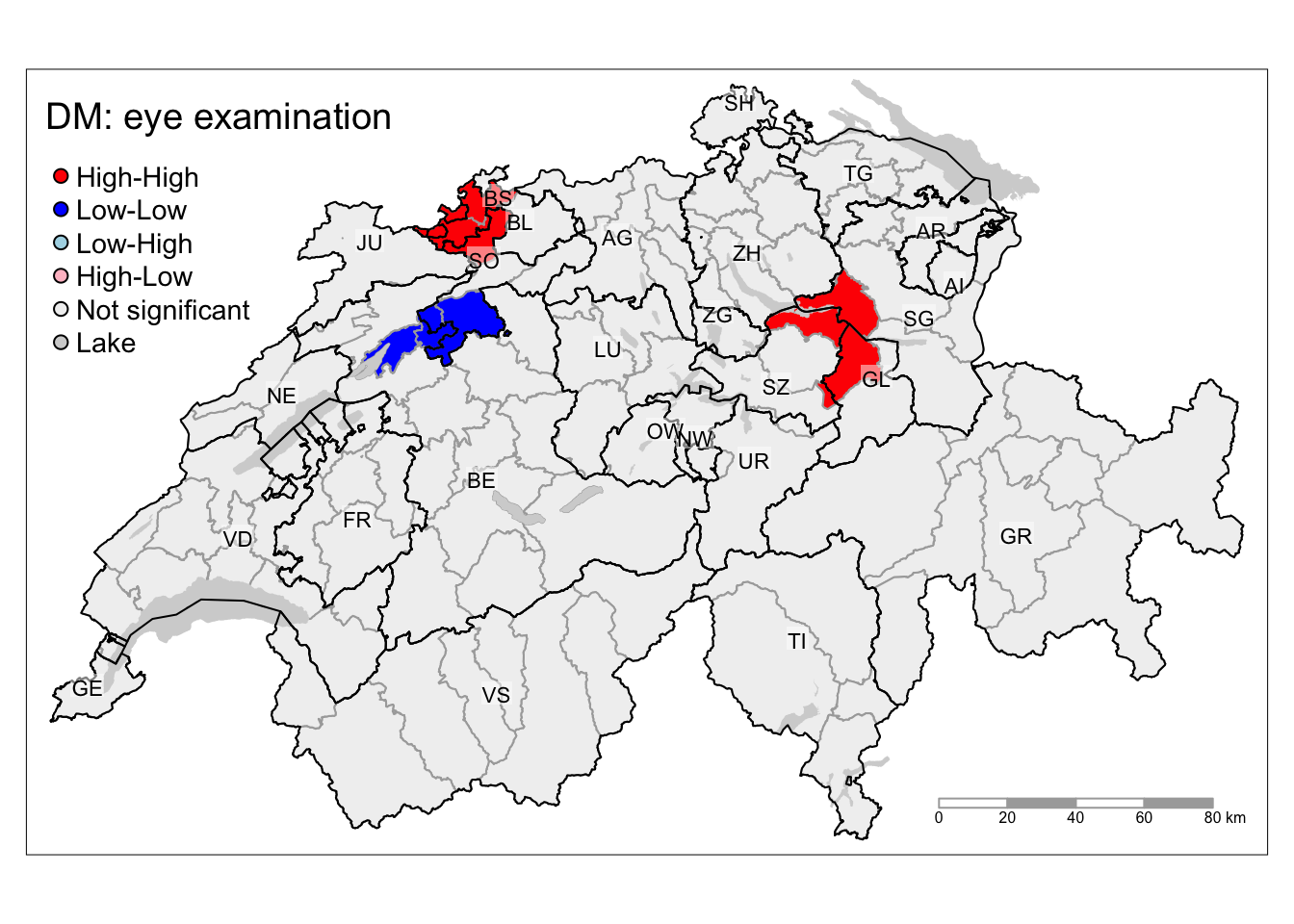 |
| 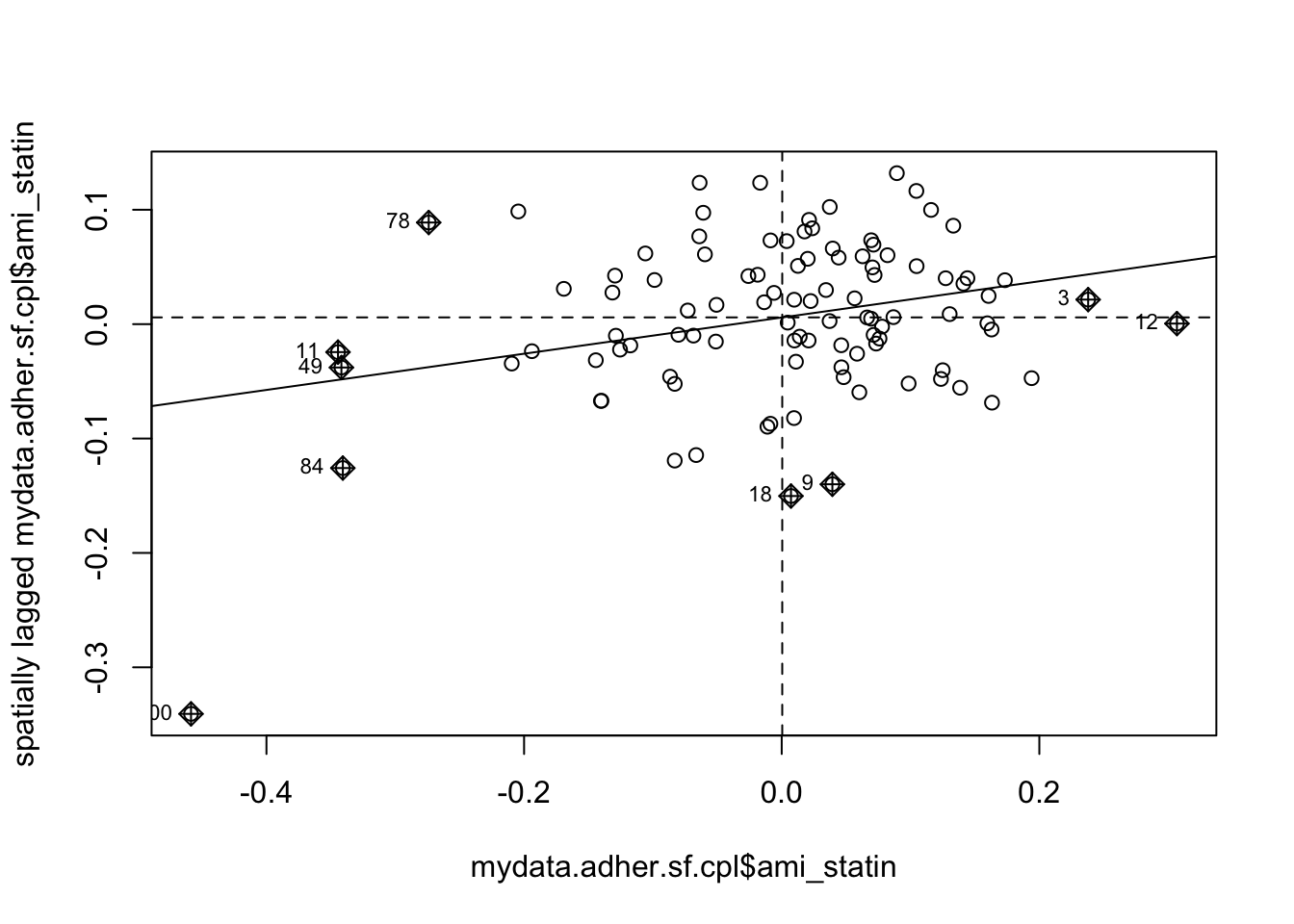 | 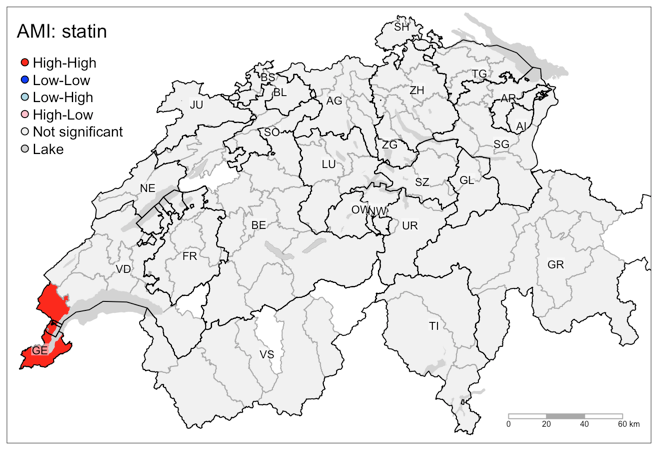 |
| 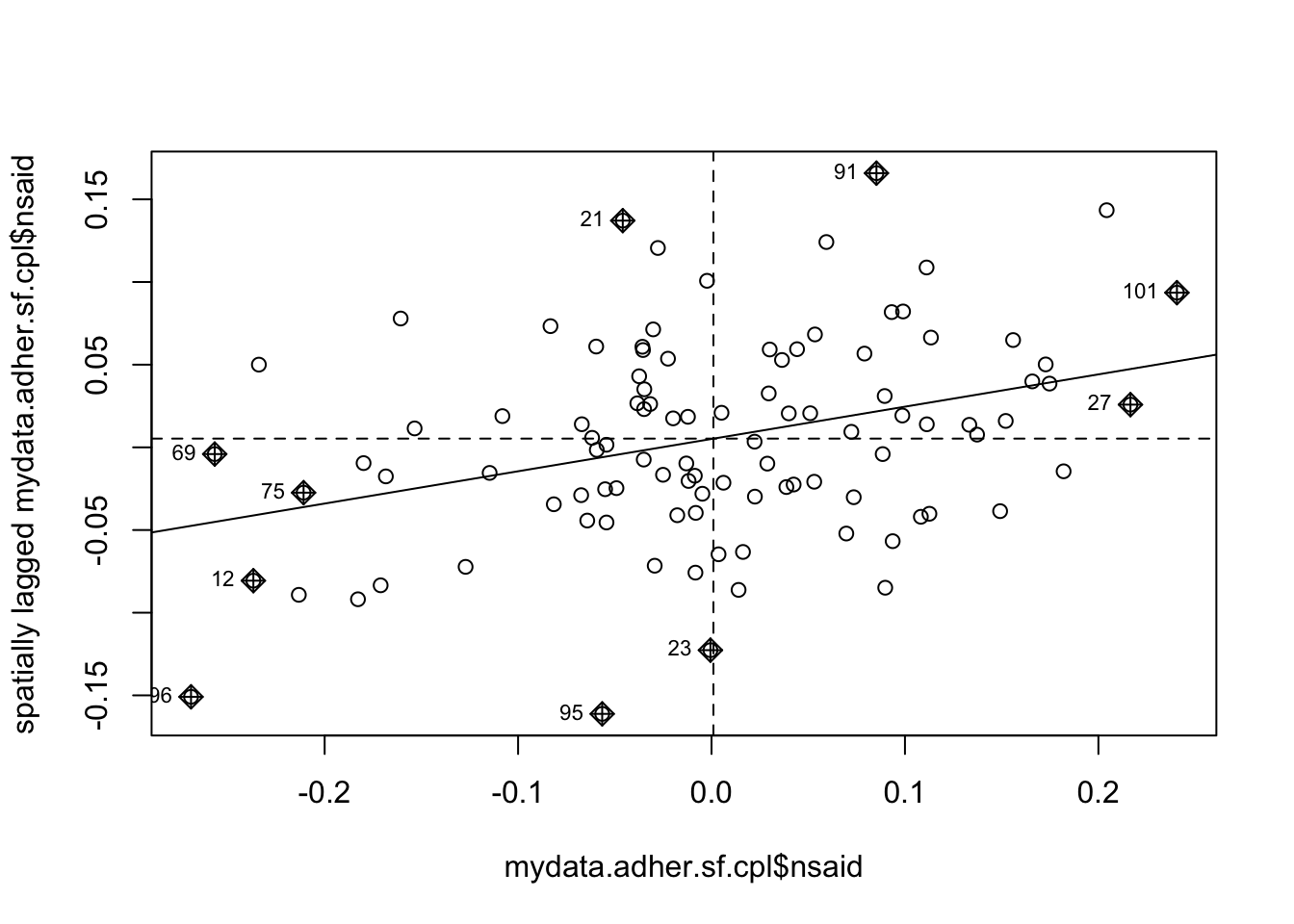 | 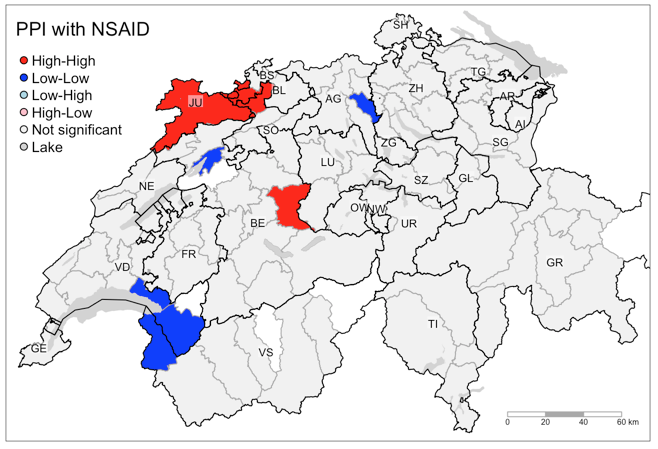 |
| 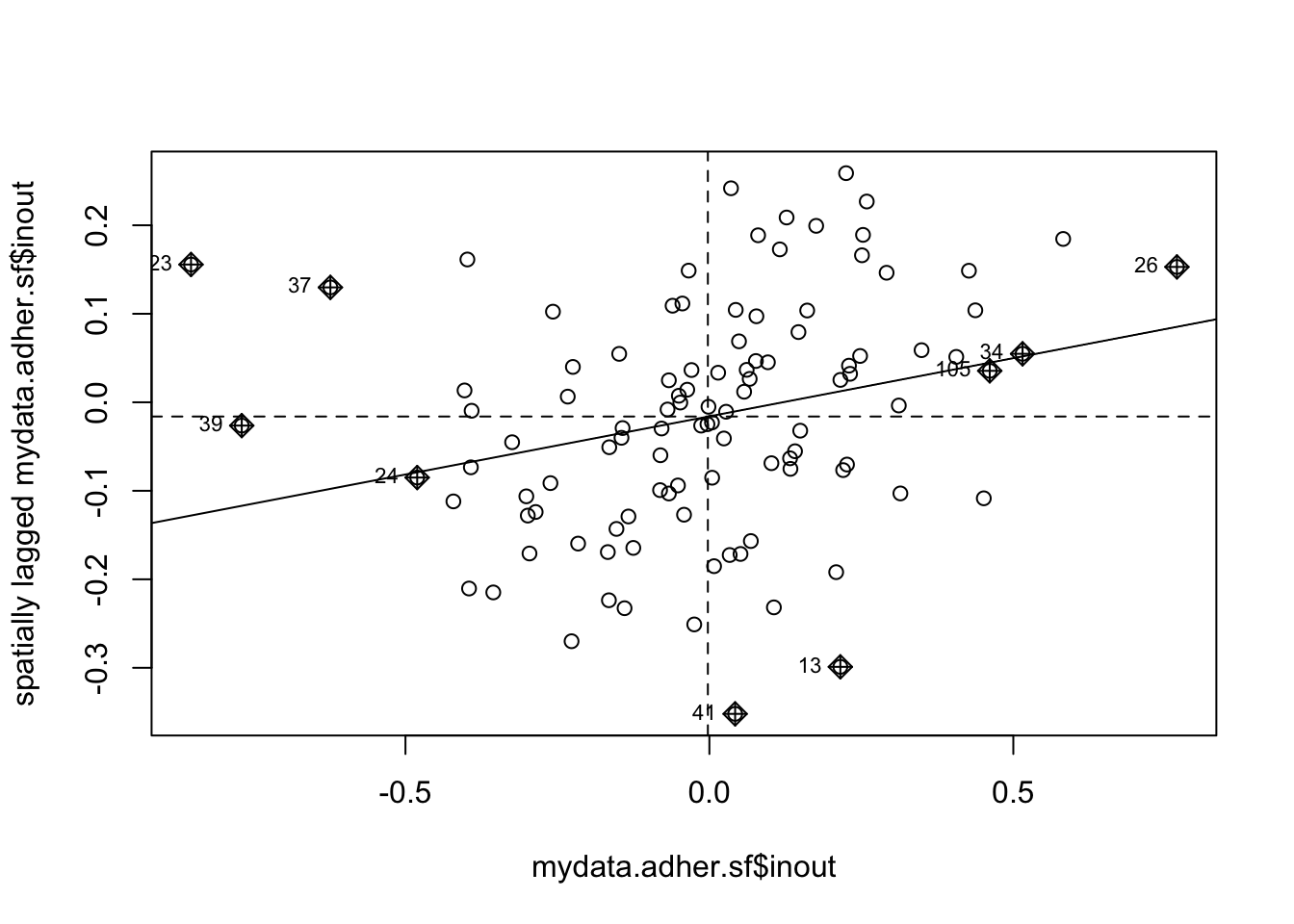 | 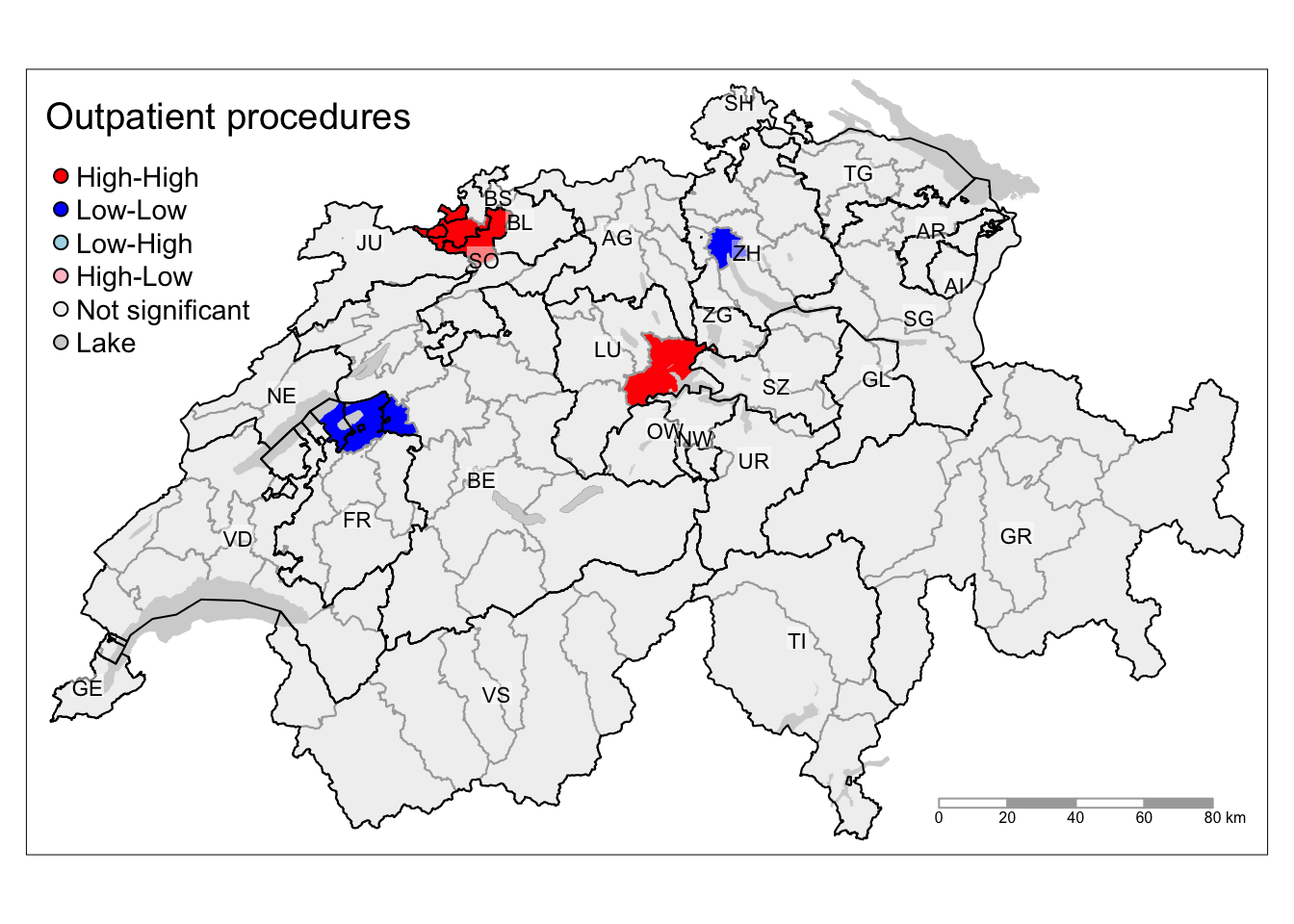 |

Figure S5: Moran scatterplots (left) and Local Indicators of Spatial Association (LISA) maps (right) for adherence patterns that had significant and very weak global Moran’s I values.
